# Acarbose reduces *Pseudomonas aeruginosa* respiratory tract infection in type 2 diabetic mice

**DOI:** 10.1186/s12931-023-02619-8

**Published:** 2023-12-14

**Authors:** Lin Liu, Haiyang Fan, Liang Li, Yunping Fan

**Affiliations:** 1 Department of Otolaryngology, The Seventh Affiliated Hospital, Sun Yat-Sen University, Shenzhen, People’s Republic of China; 2 Department of Pharmacology, School of Medicine, Southern University of Science and Technology, Shenzhen, People’s Republic of China; 3 Shenzhen Institutes of Advanced Technology, Chinese Academy of Sciences, Shenzhen, China

**Keywords:** Type 2 diabetes mellitus, *P. aeruginosa*, Respiratory tract infection, Acarbose, NF-κB signaling pathway

## Abstract

**Background:**

Type 2 diabetes mellitus (T2DM) is widely prevalent worldwide, and respiratory tract infections (RTIs) have become the primary cause of death for T2DM patients who develop concurrent infections. Among these, *Pseudomonas aeruginosa* infection has been found to exhibit a high mortality rate and poor prognosis and is frequently observed in bacterial infections that are concurrent with COVID-19. Studies have suggested that acarbose can be used to treat T2DM and reduce inflammation. Our objective was to explore the effect of acarbose on *P. aeruginosa* RTI in T2DM individuals and elucidate its underlying mechanism.

**Methods:**

High-fat diet (HFD) induction and *P. aeruginosa* inhalation were used to establish a RTI model in T2DM mice. The effect and mechanism of acarbose administered by gavage on *P. aeruginosa* RTI were investigated in T2DM and nondiabetic mice using survival curves, pathological examination, and transcriptomics.

**Results:**

We found that *P. aeruginosa* RTI was more severe in T2DM mice than in nondiabetic individuals, which could be attributed to the activation of the NF-κB and TREM-1 signaling pathways. When acarbose alleviated *P. aeruginosa* RTI in T2DM mice, both HIF-1α and NF-κB signaling pathways were inhibited. Furthermore, inhibition of the calcium ion signaling pathway and NF-κB signaling pathway contributed to the attenuation of *P. aeruginosa* RTI by acarbose in nondiabetic mice.

**Conclusions:**

This study confirmed the attenuating effect of acarbose on *P. aeruginosa* RTIs in T2DM and nondiabetic mice and investigated its mechanism, providing novel support for its clinical application in related diseases.

**Supplementary Information:**

The online version contains supplementary material available at 10.1186/s12931-023-02619-8.

## Introduction

According to the International Diabetes Federation and the World Health Organization, as of 2021, approximately one in ten people worldwide have diabetes, with a total of 537 million diabetics [1, 2]. Diabetes mellitus prevalence has been steadily increasing over the past few decades, and it is expected to continue in the future [3]. Among these, T2DM accounts for over 90% of all diabetes cases globally. It is characterized by pancreatic β-cell dysfunction, leading to insulin resistance and insufficient insulin secretion [1]. Moreover, T2DM is challenging to diagnose in the early stages, and it is often accompanied by other complications or diagnosed concurrently with other diseases [4]. In 2019, diabetes directly caused 1.5 million fatalities, and in 2021, diabetes was estimated to be responsible for 6.7 million deaths, equivalent to one death every five seconds [2].

It has been noted that the incidence and mortality rates of vascular complications of diabetes have decreased in recent years. On the other hand, the incidence and mortality rate of nonvascular and noncancer complications, including infections, has become increasingly important [5–7]. Among all complications, RTIs have nearly tripled in proportion and account for a significant proportion of nonvascular and noncancer deaths in T2DM [7, 8]. Meanwhile, numerous studies have demonstrated that diabetic patients suffering from RTIs are at significantly higher risk of hospitalization, longer hospital stays, and death than nondiabetic patients [9–16]. However, existing studies have not yet been able to determine whether diabetes is an independent risk factor for the increased incidence or severity of common RTIs [10, 14].

According to current research, diabetic patients are at increased risk and severity of RTIs caused by certain pathogens, particularly *P. aeruginosa *[14, 17–19]. As a common clinical opportunistic pathogen, *P. aeruginosa* is a significant pathogen that causes RTIs in immunocompromised patients [20, 21]. In ICU patients with *P. aeruginosa* pneumonia, diabetes was independently associated with a higher mortality rate (OR, 5.46; 95% CI, 1.05–28.42; P = 0.04) [22]. In mouse models, diabetic mice had a higher bacterial load in their lung homogenates after RTI with *P. aeruginosa* than nondiabetic control mice [23, 24]. Individuals with T2DM are more susceptible to *P. aeruginosa* RTIs, which are characterized by drug resistance, long-term treatment, and poor prognosis, resulting in negative health and economic impacts [25–27]. Additionally, *P. aeruginosa* is a common pathogen in COVID-19 patients with secondary infections [28–31].

Therefore, the current COVID-19 outbreak may potentially increase the risk of mixed and secondary infections, resulting in serious consequences for diabetic patients who are already struggling with *P. aeruginosa* infections. It has been observed that acarbose use could improve the survival rate of T2DM patients with COVID-19 [32, 33]. Moreover, acarbose treatment has been demonstrated to reduce serum inflammatory cytokines and alleviate the chronic inflammatory state in diabetic patients [34, 35]. Research also suggests that acarbose can control infection independently of blood glucose levels and has the potential to be a therapeutic drug in such cases [32, 36, 37].

In conclusion, the treatment of *P. aeruginosa* RTIs in T2DM individuals is a compelling and challenging task. Acarbose, as the most widely used α-glucosidase inhibitor in clinical T2DM treatment [38–40], holds significant research value in terms of its potential broad-spectrum anti-infective effects and its specific mechanisms in combating RTIs in diabetes patients. However, current research in this area still revolves around simple clinical data collection. Research on the specific mechanisms, immune changes, and inflammatory signaling pathways that exacerbate *P. aeruginosa* RTI in patients with T2DM and the role of acarbose treatment in this complex condition remains limited.

The objective of this study was to establish mouse models of *P. aeruginosa* RTI in both T2DM and nondiabetic mice, which were orally administered acarbose. Using pathological and transcriptomic techniques, our goal was to analyze the complex disease condition of T2DM combined with *P. aeruginosa* RTI and to investigate the effects of acarbose on these infections.

## Method

### Animals

C57BL/6 J mice (6 weeks old, male, body weight 18–22 g) were obtained from Charles River Laboratories (Guangzhou, China). The mice were acclimatized for one week and then randomly assigned to groups with similar average body weights. During the study, the mice were housed in individual ventilated cages under specific pathogen-free (SPF) conditions, with a temperature maintained at 21 ± 2 °C and 55 ± 15% relative humidity, and a 12-h light/dark cycle. All animal experiments were performed in accordance with the guidelines of the Technological Commission of The People's Republic of China on the protection of animals used for scientific purposes and were approved by the Animal Laboratory of Shenzhen Institute of Advanced Technology, Chinese Academy of Sciences (Shenzhen, China) under the certificate of Application for the Use of Animals (approval ID: SIAT-IACUC-20211201-YYS-DXSZZX-LL-A2056-01).

### T2DM mouse model

After acclimatization to the new surroundings, T2DM was induced in C57BL/6 J mice by feeding half of them ad libitum with a HFD: Rodent Diet with 45 kcal% Fat (D12451, Research Diets Inc) [41–44]. The other half were fed a conventional diet as the control group. Both groups were maintained on their respective diets for 12 weeks and body weights were recorded every 3 weeks. After this study period, blood was collected from the tail-tip vein of the mice after a 3-h fast to measure blood glucose concentration using a blood glucose meter (Roche, Swiss Confederation) and blood glucose test strips (Roche, Swiss Confederation). Only mice with a fasting blood glucose level ≥ 11.1 mmol/l on an HFD diet were included in the T2DM group, with a success rate of approximately 70%.

At the end of the comparative feeding process, OGTT was assessed in control and HFD-fed mice after 6 h of fasting. At 0 min, blood was collected from the tail-tip vein to measure blood glucose concentration using a blood glucose meter and blood glucose test strips. Glucose at a dose of 4 g/kg BW was administered orally by gavage simultaneously. After glucose gavage, blood glucose concentrations were determined at 15, 30, 60, 90, 120 and 180 min.

### Acarbose treatment

To investigate the potential role of acarbose (Acarbose Hydrate, Aladdin, ≥ 98%, Shanghai, China) in *P. aeruginosa* RTI mice, we randomly divided the T2DM mice and the control mice into four groups and administered them by oral gavage. The four groups were the sterile water-treated control group, the sterile water-treated T2DM group, the acarbose-treated control group, and the acarbose-treated T2DM group.

Acarbose was administered via gavage at a daily dose of 40 mg/kg/day, dissolved in sterile water (2 mg/ml). Equal volumes of acarbose solution and sterile water were administered at all times. These treatments were given for two weeks and continued following *P. aeruginosa* infection.

### *P. aeruginosa* infection

All mice were anaesthetized with avertin (4 µg/200 µl/20 g, Sigma, USA). Half of the mice were randomly selected from each of the four aforementioned groups and inhaled 40 μl of 1*10^6^ colony forming units (CFU)/ml *P. aeruginosa* (PAO1 ATCC) [45] through the trachea, while the remaining mice were given an equivalent volume of PBS as the control. After inhalation, the mice were returned to their cages and monitored until they regained consciousness. All *P. aeruginosa* related experiments were conducted in BSL-2 (including ABSL-2) of the Shenzhen Institute of Advanced Technology, Chinese Academy of Sciences.

The animals were then categorized into eight groups based on the aforementioned classifications. These groups were: PBS-Water-Ctrl (Ctrl), PBS-Acarbose-Ctrl (Ctrl + Acarbose), PBS-Water-T2DM (Diabetes), PBS-Acarbose-T2DM (Diabetes + Acarbose), PAO1-Water-Ctrl (Ctrl + Infected), PAO1-Acarbose-Ctrl (Ctrl + Acarbose + Infected), PAO1-Water-T2DM (Diabetes + Infected), and PAO1-Acarbose-T2DM (Diabetes + Acarbose + Infected).

### Sample collection and processing

The study endpoint was the fourth day following *P. aeruginosa* infection. On this day, the mice were anaesthetized with avertin (4 µg/200 µl/20 g, Sigma, USA) and euthanized by exsanguination. The mice’s lungs were rapidly dissected and removed. The left lung was isolated, washed in PBS, and fixed in 4% paraformaldehyde. The remaining lungs were snap-frozen in liquid nitrogen and stored at − 80 °C for future analysis. The mice' well-being was monitored by recording their body weights daily throughout the acarbose treatment and the trial.

### Pathological histology

To conduct histological analyses, the left lungs were fixed in 4% paraformaldehyde for 48 h. Then they were dehydrated, embedded in paraffin, and sectioned into 5 µm slices. Afterwards, each group's lung tissue sections were stained with hematoxylin and eosin (H&E) and examined under light microscopy (Nikon, Tokyo, Japan) to assess morphological changes, identify histopathological features, and distinguish inflammatory cells.

### RNA isolation and sequencing

Total RNA was extracted from frozen lung tissue using TRIzol reagent (Invitrogen, CA, USA) following the manufacturer's protocol. RNA purity and quantity were determined with a NanoDrop 2000 Spectrophotometer (Thermo Scientific, USA), and RNA integrity was assessed with an Agilent 2100 Bioanalyzer (Agilent Technologies, Santa Clara, CA, USA). The VAHTS Universal V6 RNA-Seq Library Prep Kit was employed to build libraries following the manufacturer's instructions. The libraries were then sequenced on an Illumina NovaSeq 6000 platform, producing 150 bp paired-end reads. Transcriptome sequencing was conducted on the aforementioned frozen lung tissue, stored at − 80 °C, using the Illumina sequencing platform.

### Transcriptome data analysis

Fastp [46] was used to eliminate low-quality reads and obtain clean reads from raw reads. The clean reads were mapped to the mouse genome (Gene Database, GRCm39) utilizing HISAT2 [47]. HTSeq-count [48] was used to calculate FPKM [49] and read counts for each gene. Subsequently, DESeq2 [50] was utilized to perform differential expression analysis, with Q value < 0.05 and significant change difference (|log2(fold change)|> 1.5) as the threshold for identifying differentially expressed genes (DEGs).

Hierarchical cluster analysis was performed on the DEGs to examine expression patterns across different groups. The results were displayed as volcano plots to show upregulated or downregulated DEGs. Furthermore, to provide detailed information on the altered functions and pathways, Gene Ontology (GO) and Kyoto Encyclopedia of Genes and Genomes (KEGG) pathway enrichment analyses of DEGs were conducted using a statistical analysis based on the hypergeometric distribution. The transcriptome sequencing and analysis described above were performed by OE Biotech (Shanghai, China). Specific result diagrams were generated using R and accessed at http://www.bioinformatics.com.cn (v 3.2.0).

### Ingenuity pathway analysis (IPA)

Based on the Ingenuity Knowledge Base, IPA software (QIAGEN, Germany) was used to analyse DEGs and visualize the biological functions of various molecules and their network of interactions. DEGs from each experiment were imported and analysed by IPA to reveal the underlying mechanisms of the observed phenomenon among the aforementioned eight groups. In addition to the Canonical pathways, upstream regulator analysis and downstream effect analysis were conducted concurrently. IPA filter settings were limited to the mouse, and the analytical results were filtered based on the P-value and then reported as a Z score.

### Verification of DEGs by qPCR

Genes were selected for quantitative polymerase chain reaction (qPCR) verification of DEGs (Additional file 2: Table S1). Primers were synthesized by Sangon Biotech (Shanghai, China) based on published murine sequences from GenBank (Additional file 2: Table S1). Total RNA was extracted with TRIzol (Invitrogen, CA, USA) and transcribed with a Prime ScriptTM RT reagent kit (Takara, Japan). qPCR was performed using Prime Script® RT Master Mix (Takara, Japan), with each reaction conducted in triplicate. The relative changes in gene expression were normalized by the internal reference housekeeper gene 18S and counted using the 2^−ΔΔCT^ method.

### Statistical analysis

The data are presented as the mean ± SEM, and all quantitative experiments were independently repeated with at least three biological replicates. Statistical analyses were performed using SPSS 19.0 software (Chicago, IL, USA) and GraphPad Prism 8.0.2 (San Diego, CA, USA). A P value < 0.05 was considered statistically significant, while values below 0.1 were considered “suggestive”.

## Results

### HFD-induced T2DM mouse model

**Fig. 1:**
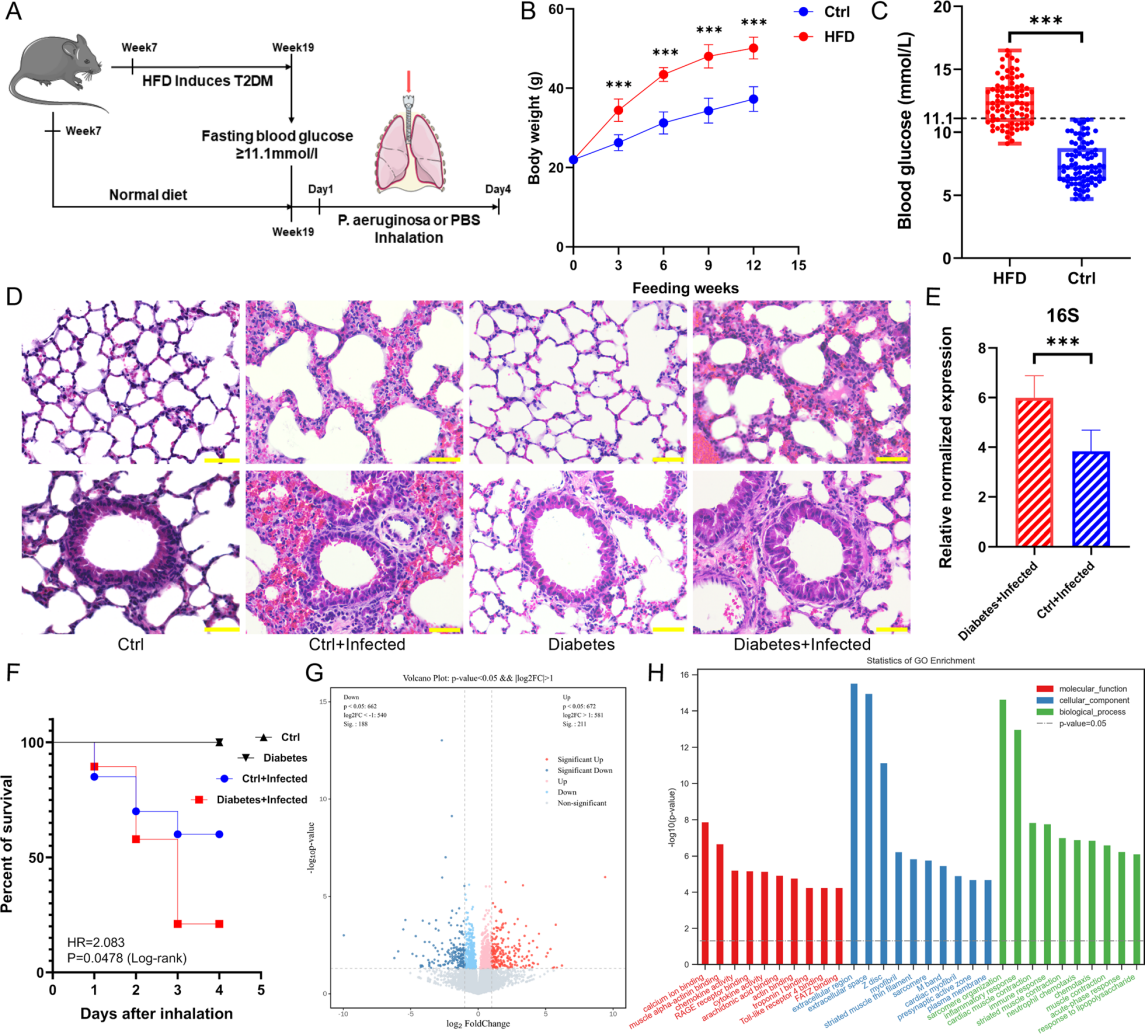
**A** Graphic illustration of the experimental design of *P. aeruginosa* respiratory infection in the T2DM mouse model and control mice. **B** The body weights of HFD-induced and control mice (n = 85, ***P < 0.005). **C** The fasting blood glucose of HFD-induced and control mice (n = 85, ***P < 0.005). **D** H&E staining of lung tissue sections from mice in the Ctrl, Diabetes, Ctrl + Infected, and Diabetes + Infected groups (scale bar = 50 µm). **E** The expression of *P. aeruginosa* in the lung tissue of mice in the Diabetes + Infected and Ctrl + Infected groups (n = 5, ***P < 0.005). **F** Kaplan‒Meier survival curve of the Ctrl, Diabetes, Ctrl + Infected, and Diabetes + Infected groups four days after inhalation. **G** Volcano plot of DEGs between the Ctrl + Infected, and Diabetes + Infected groups (Q < 0.05; |log2(fold change)|> 1). **H** Bar chart for GO functional enrichment of DEGs

In this study, we employed a HFD to induce T2DM in mice and established both T2DM and non-diabetic mouse models infected with *P. aeruginosa* in the respiratory tract (Fig. 1A, Additional file 1: Fig. S1).

Body weight measurements were taken every three weeks during the 12-week feeding period. They showed significant different changes between the HFD-fed mice and the control mice. Although there was no significant difference in weight between the two groups initially, at the end of the 12-week feeding period, the average weight of the HFD-induced mice was 50.15 ± 1.39 g (mean ± SEM), which was significantly higher than the average weight of the control group of 37.27 ± 1.54 g (mean ± SEM), indicating severe obesity (Fig. 1B).

The fasting blood glucose levels of the mice were measured by tail vein blood sampling, and the results showed that the average blood glucose level of mice fed the HFD for 12 weeks was 12.41 ± 0.19 mmol/L (mean ± SEM), which was significantly higher than the average blood glucose level of the control group of 7.48 ± 0.18 mmol/L (mean ± SEM) (Fig. 1C). There are significant differences in OGTT results between HFD-fed mice and control mice (Additional file 1: Fig. S3). Out of the 85 mice fed HFD, 62 (72.94%) had a fasting blood glucose level exceeding 11.1 mmol/L at 12 weeks of feeding and were considered appropriate T2DM mouse models for the subsequent experiments.

### *P. aeruginosa* respiratory tract infection in T2DM mice is significantly more severe than that in control mice

In this study, we investigated the effect of *P. aeruginosa* infection on mice with and without T2DM. Following inhalation of *P. aeruginosa*, the average body weight of the Diabetes + Infected group decreased significantly from 51.95 to 48.64 g (n = 10, P < 0.05). Similarly, the Ctrl + Infected group’s body weight was also significantly reduced from 36.22 to 33.32 g (n = 10, P < 0.05). Inhalation of *P. aeruginosa* through the trachea caused weight loss in both groups. However there was no significant difference in the degree of weight loss between the Diabetes + Infected and Ctrl + Infected groups (Additional file 1: Fig. S4A, B).

Obviously, according to the Kaplan‒Meier survival curve, the Diabetes + Infected group had a significantly lower four-day survival rate (21.05%) than the Ctrl + Infected group (60%). The hazard ratio[51] (HR, Diabetes + Infected VS Ctrl + Infected) was 2.083, indicating that the risk of death caused by *P. aeruginosa* infection was higher in T2DM mice than in the control group (Fig. 1F).

Moreover, H&E staining of lung tissue sections revealed significant pathological signs of inflammation induced by *P. aeruginosa* infection in both the Diabetes + Infected and Ctrl + Infected groups, including inflammatory cell infiltration, alveolar damage, and fusion, thickening of the alveolar septum and airway walls, and irregular arrangement of airway epithelial cells (Fig. 1D). Furthermore, qPCR analysis revealed significantly higher expression of *P. aeruginosa* in the lung tissue of the Diabetes + Infected group than in that of the Ctrl + Infected group (Fig. 1E).

### *P. aeruginosa* respiratory tract infection in T2DM mice is significantly more severe than that in control mice

RNA-seq analysis identified a significant number of DEGs between the Diabetes + Infected and Ctrl + Infected groups, with 188 downregulated and 211 upregulated DEGs (P < 0.05, |log2(fold change)|> 1) (Fig. 1G). We conducted functional classification and enrichment analysis of the DEGs using Gene Ontology (GO) and presented the results in a histogram, which was categorized into three parts: molecular function (MF), cellular component (CC), and biological process (BP) (Fig. 1H).

**Fig. 2:**
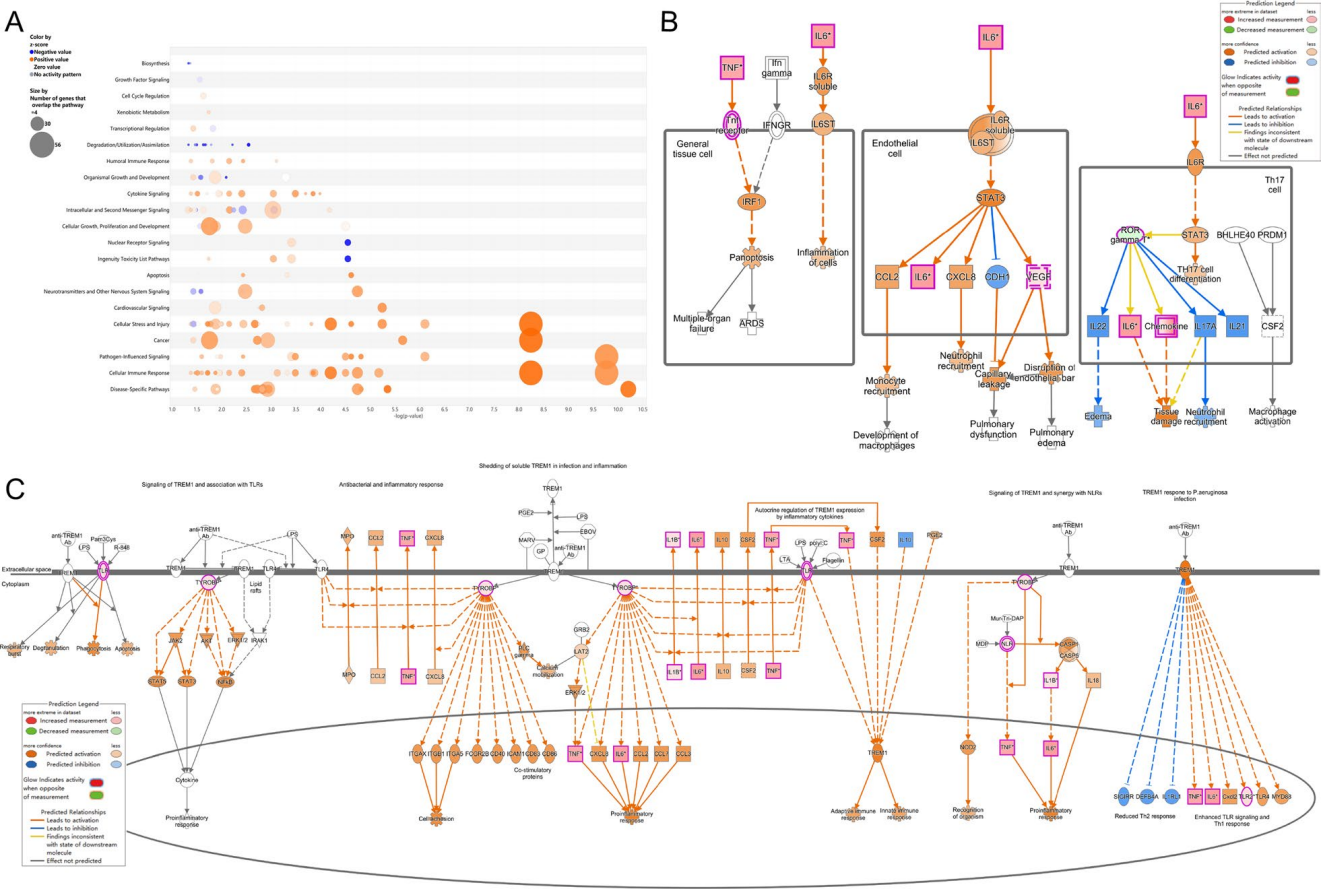
**A** Bubble chart of the canonical pathways from IPA analysis DEGs (P < 0.05; |z-score|≥ 0). Z-scores reported whether a pathway was activated (z-score > 0) or inhibited (z-score < 0) in the Diabetes + Infected compared to Ctrl + Infected. **B** ,  **C** Signaling pathway of **B** Pathogen-induced cytokine storm and **C** TREM1 were activated in Diabetes + Infected compared to Ctrl + Infected (green: down-regulated; red: up-regulated; blue: predicted to inhibit; orange: predicted to activate)

IPA software analysis of the DEGs (Diabetes + Infected VS Ctrl + Infected) showed that after *P. aeruginosa* infection, T2DM individuals exhibited more changes in canonical pathways that promoted inflammation and increased infection severity, including cellular stress and damage, cytokine signaling, and pathogen-induced signaling (Fig. 2A).

**Fig. 3:**
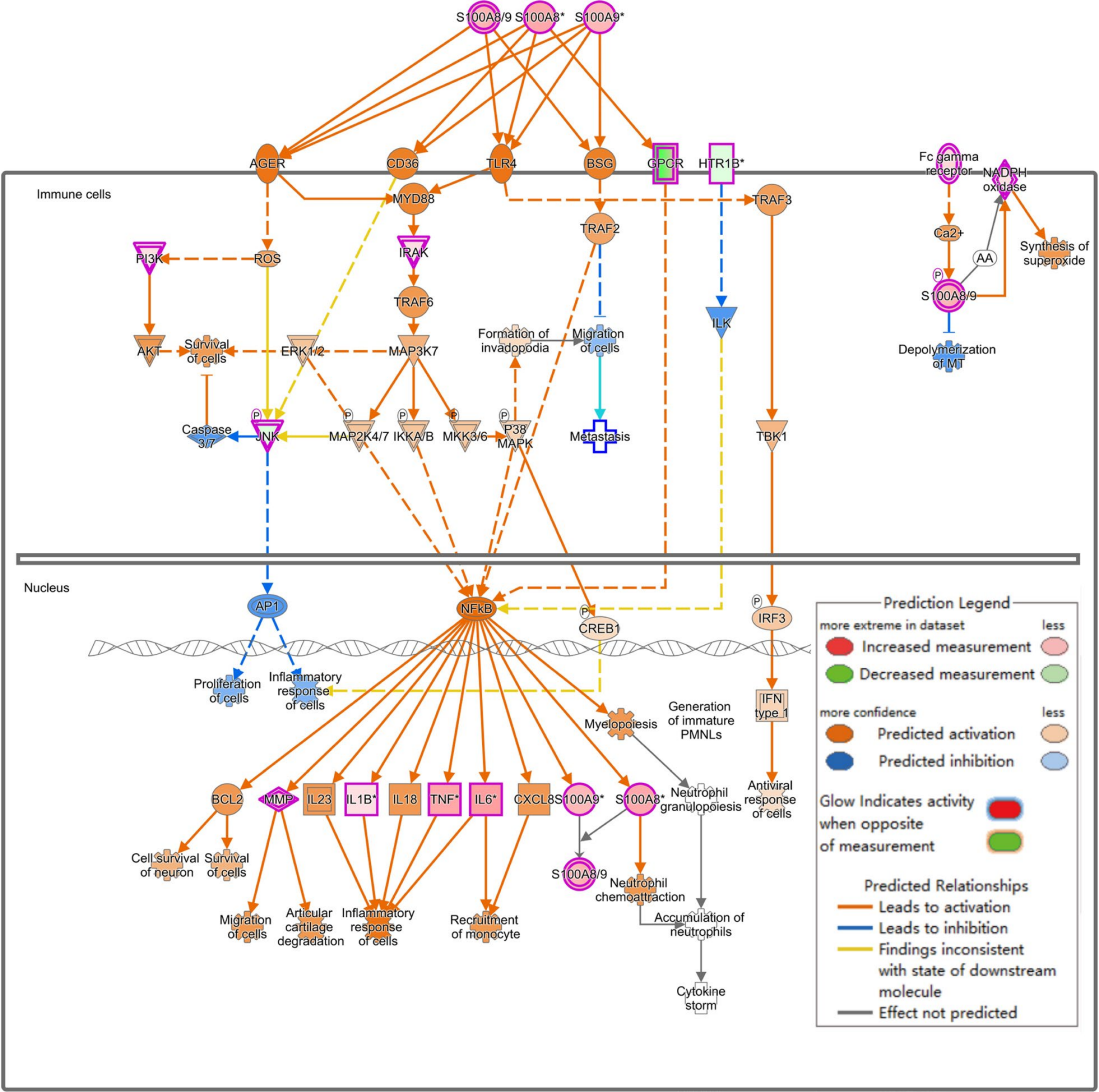
NF-κB signaling pathway was activated in Diabetes + Infected compared to Ctrl + Infected (green: down-regulated; red: up-regulated; blue: predicted to inhibit; orange: predicted to activate)

The activation of the pathogen-induced cytokine storm signaling pathway (Z score = 4.110) (Fig. 2B), based on upregulated expression of IL-6 and TNF, along with the activation of the TREM-1 signaling pathway (Z score = 3.162) (Fig. 2C) and the S100 family NF-κB signaling pathway (Z score = 4.276) (Fig. 3), which were upregulated due to overexpression of TNF, IL-1β, and IL-6, may have contributed significantly to the observed phenotypes.

In addition, classical pathway analysis revealed regulation changes in the HIF-1α signaling pathway (Z score = 3.130) and calcium signaling pathway (Z score = − 4.276). Taken together, the regulation of these pathways collectively mediates the exacerbation of *P. aeruginosa* RTI in T2DM. Figure S5A illustrates that the qPCR detection results of the mentioned genes were consistent with the RNA-seq results.

### Acarbose reduces *P. aeruginosa* infection in T2DM mouse lungs

**Fig. 4:**
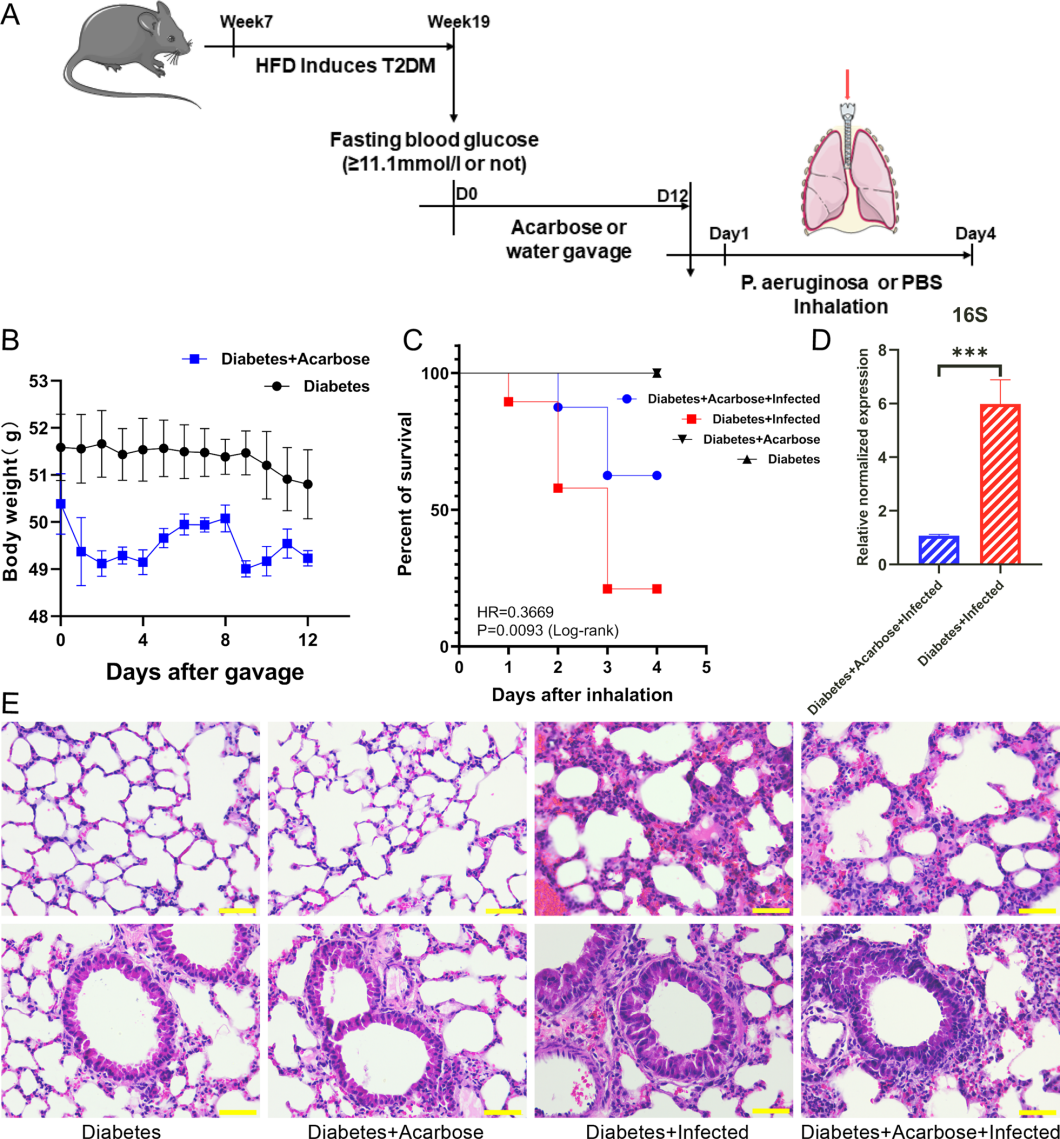
**A** Schematic representation of the experimental design of acarbose gavage and *P. aeruginosa* infection in the T2DM mouse model. **B** Body weights during acarbose treatment in the Diabetes and Diabetes + Acarbose groups (n = 20). **C** Survival curve of the Diabetes and Diabetes + Acarbose, Diabetes + Infected, and Diabetes + Acarbose + Infected groups four days after inhalation. **D** The expression of *P. aeruginosa* in the lung tissue of mice in the Diabetes + Infected and Diabetes + Acarbose + Infected groups (n = 5, ***P < 0.005). **E** H&E staining of lung tissue sections from mice in the Diabetes, Diabetes + Acarbose, Diabetes + Infected, and Diabetes + Acarbose + Infected groups (scale bar = 50 µm)

Alleviating the inflammatory response is the target of treating T2DM complicated by infection. To determine the potential effect of acarbose treatment on *P. aeruginosa* RTI in T2DM mice, acarbose gavage was initiated twelve days before intratracheal inhalation of *P. aeruginosa* (PAO1) (Fig. 4A, Additional file 1: Fig. S2). Twelve days of acarbose treatment did not significantly affect T2DM mice’s body weight (Fig. 4B). Both acarbose-untreated and acarbose-treated T2DM mice displayed similar weight loss after *P. aeruginosa* inhalation (Additional file 1: Fig. S4C, D).

Survivorship analysis indicated that the four-day survival rate of the Diabetes + Acarbose + Infected group was 62.5%, which was significantly higher than the 21.05% survival rate of the Diabetes + Infected group. Acarbose treatment reduced the mortality rate of *P. aeruginosa* infection in T2DM mice, as demonstrated by the HR (Diabetes + Acarbose + Infected VS Diabetes + Infected, 0.3669) (Fig. 4C).

Furthermore, histological examination of mouse lung sections stained with H&E revealed that *P. aeruginosa* infection resulted in a marked inflammatory response, lung injury, and pulmonary hemorrhage. The same phenomenon was observed in both the Diabetes + Acarbose + Infected and Diabetes + Infected groups. After acarbose treatment, these pathological changes in the Diabetes + Acarbose + Infected group mice were relatively mild (Fig. 4E). Furthermore, qPCR detection results showed that acarbose treatment significantly reduced the expression of *P. aeruginosa* in the lung tissue of the Diabetes + Acarbose + Infected group mice (Fig. 4D). These findings suggest that acarbose may ameliorate the severity of *P. aeruginosa* -induced RTI in T2DM mice.

### Acarbose reduces *P. aeruginosa* infection in T2DM mouse lungs

**Fig. 5:**
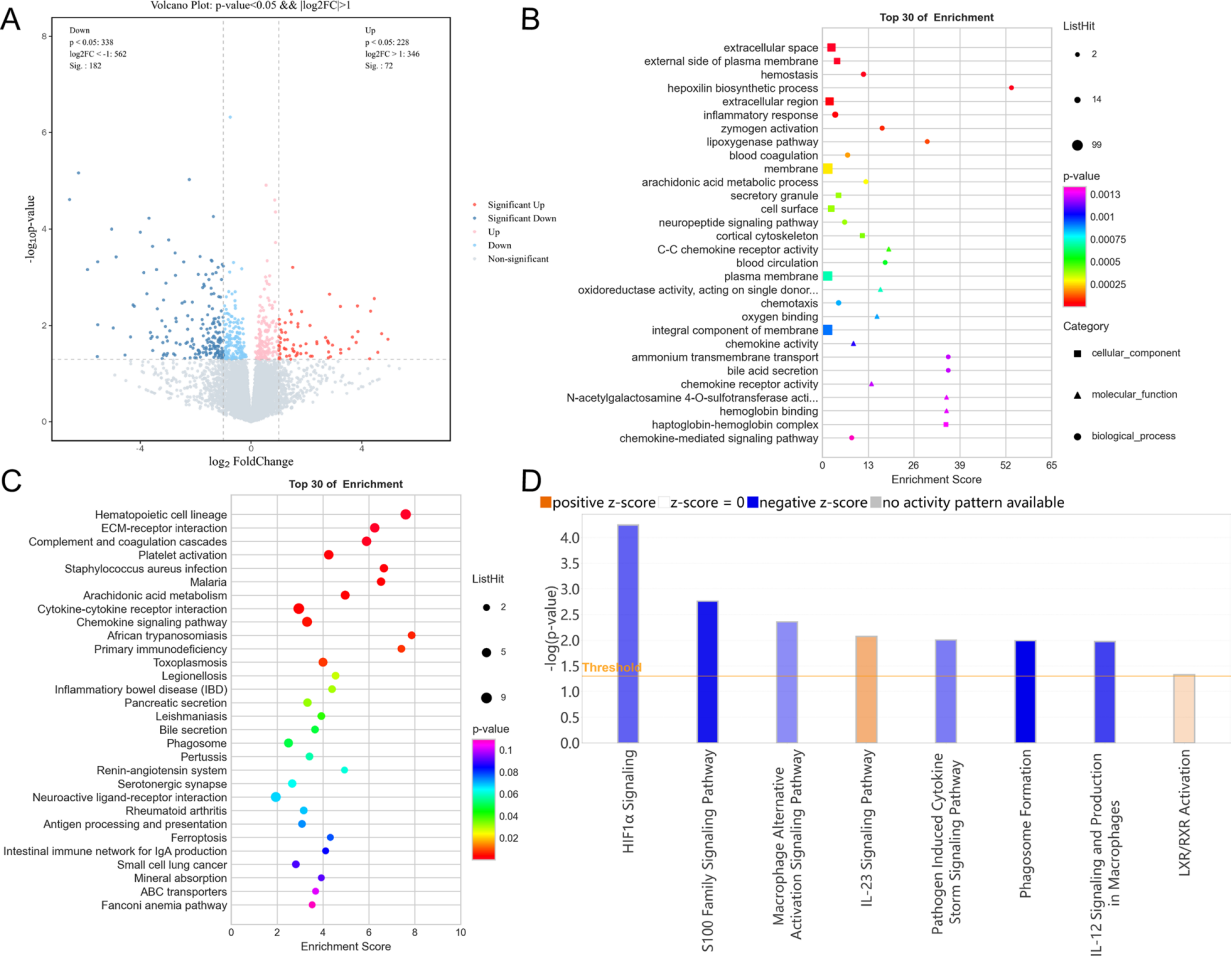
**A** Volcano plot of DEGs between the Diabetes + Acarbose + Infected and Diabetes + Infected groups (Q < 0.05; |log(fold change)|> 1). **B** Bubble chart from GO functional enrichment of DEGs between the Diabetes + Acarbose + Infected and Diabetes + Infected groups. **C** KEGG pathway analysis and enrichment of DEGs between the Diabetes + Acarbose + Infected and Diabetes + Infected groups. **D** Bar chart created by using IPA software to analyse the canonical pathways of DEGs between the Diabetes + Acarbose + Infected and Diabetes + Infected groups (P < 0.05; |Z score|≥ 0)

Transcriptome sequencing analysis showed that there were 254 DEGs between the Diabetes + Acarbose + Infected and Diabetes + Infected groups, comprising 182 downregulated genes and 72 upregulated genes (Fig. 5A). The GO annotation database analysis of these DEGs revealed significant enrichment in functions related to chemokines, cell membrane, and lipid metabolism (Fig. 5B). Moreover, KEGG analysis showed significant enrichment in pathways related to hematopoietic cell lineage, extracellular matrix-receptor interaction, complement and coagulation cascades, primary immunodeficiency, antigen processing and presentation, and ferroptosis (Fig. 5C).

**Fig. 6:**
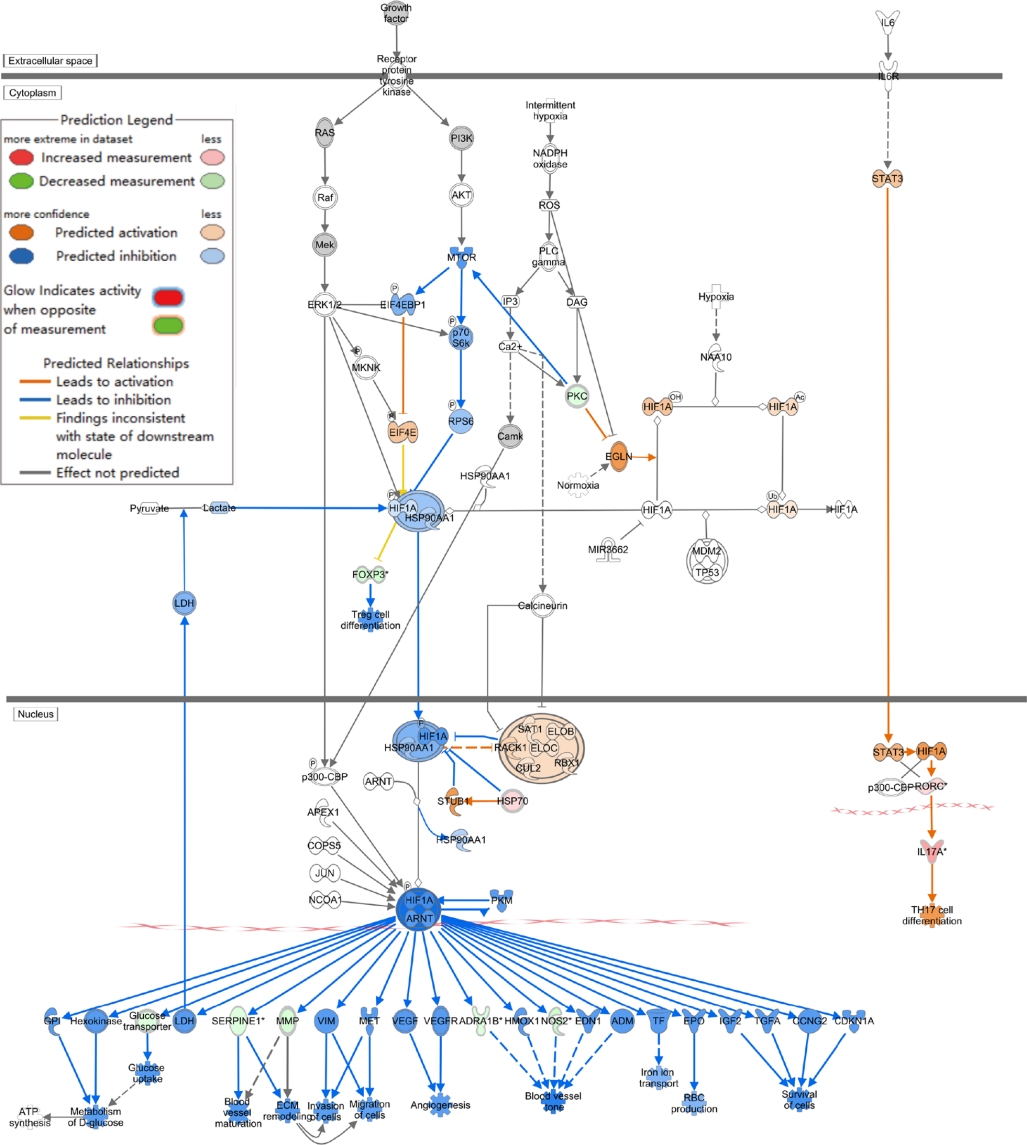
HIF-1α signaling pathway was inhibited in Diabetes + Acarbose + Infected compared to the Diabetes + Infected group (green: down-regulated; red: up-regulated; blue: predicted to inhibit; orange: predicted to activate)

Using IPA software, further analysis of the DEGs revealed the regulation of canonical pathways, including the activation of the IL-23 signaling pathway (Z score = 1.000). Conversely, HIF-1α signaling (Z score = − 1.387), the S100 family (Z score = − 2.041), and the pathogen-induced cytokine storm signaling pathway (Z score = − 1.155) were inhibited (Fig. 5D). The inhibition of the HIF-1α signaling pathway was attributed to the downregulation of NOS2 expression and upregulation of IL-17 expression, which may affect glucose uptake, extracellular matrix remodelling, cell invasion, and iron ion transport (Fig. 6).

**Fig. 7:**
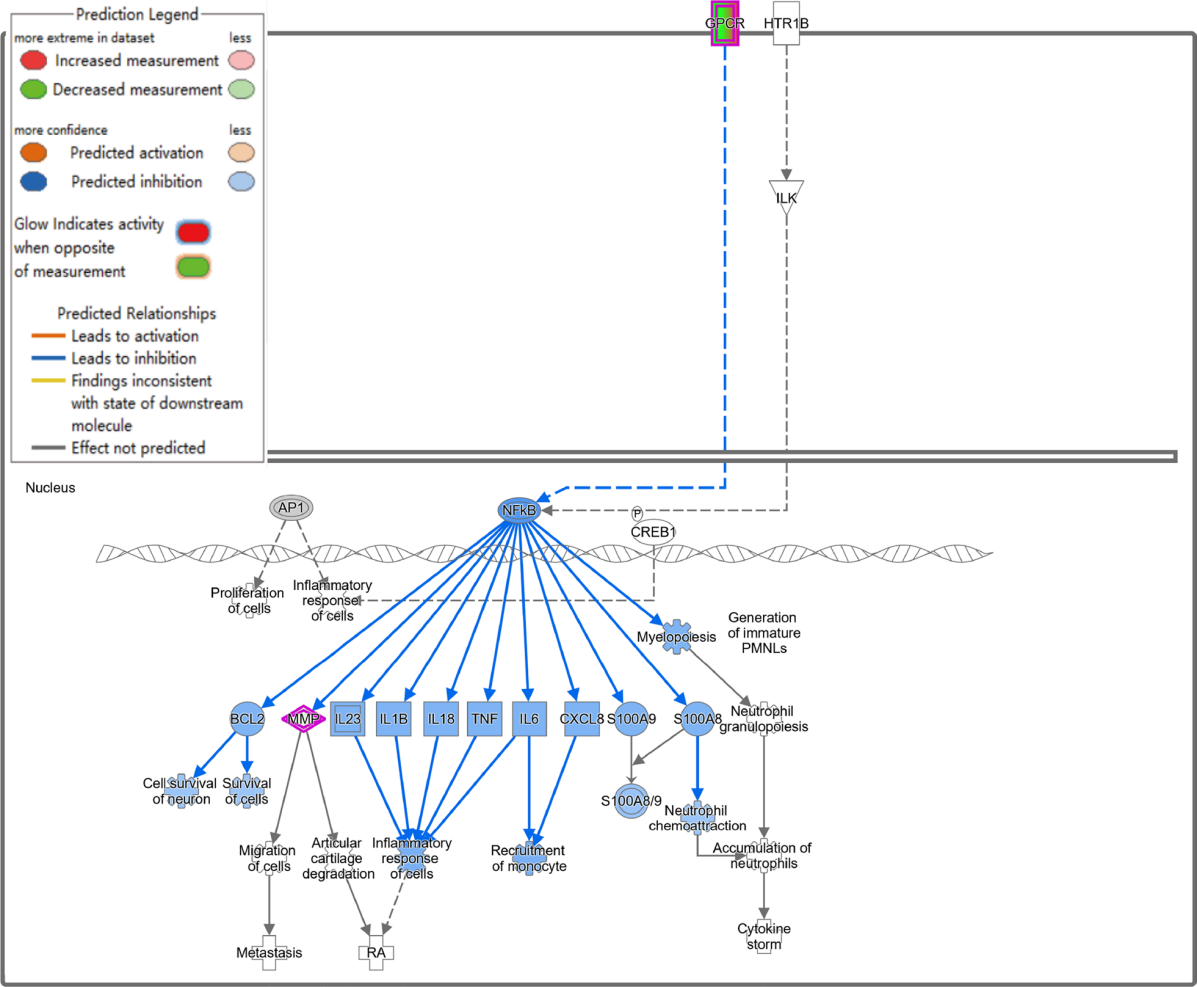
NF-κB signaling pathway was inhibited in Diabetes + Acarbose + Infected compared to the Diabetes + Infected group (green: down-regulated; red: up-regulated; blue: predicted to inhibit; orange: predicted to activate)

**Fig. 8:**
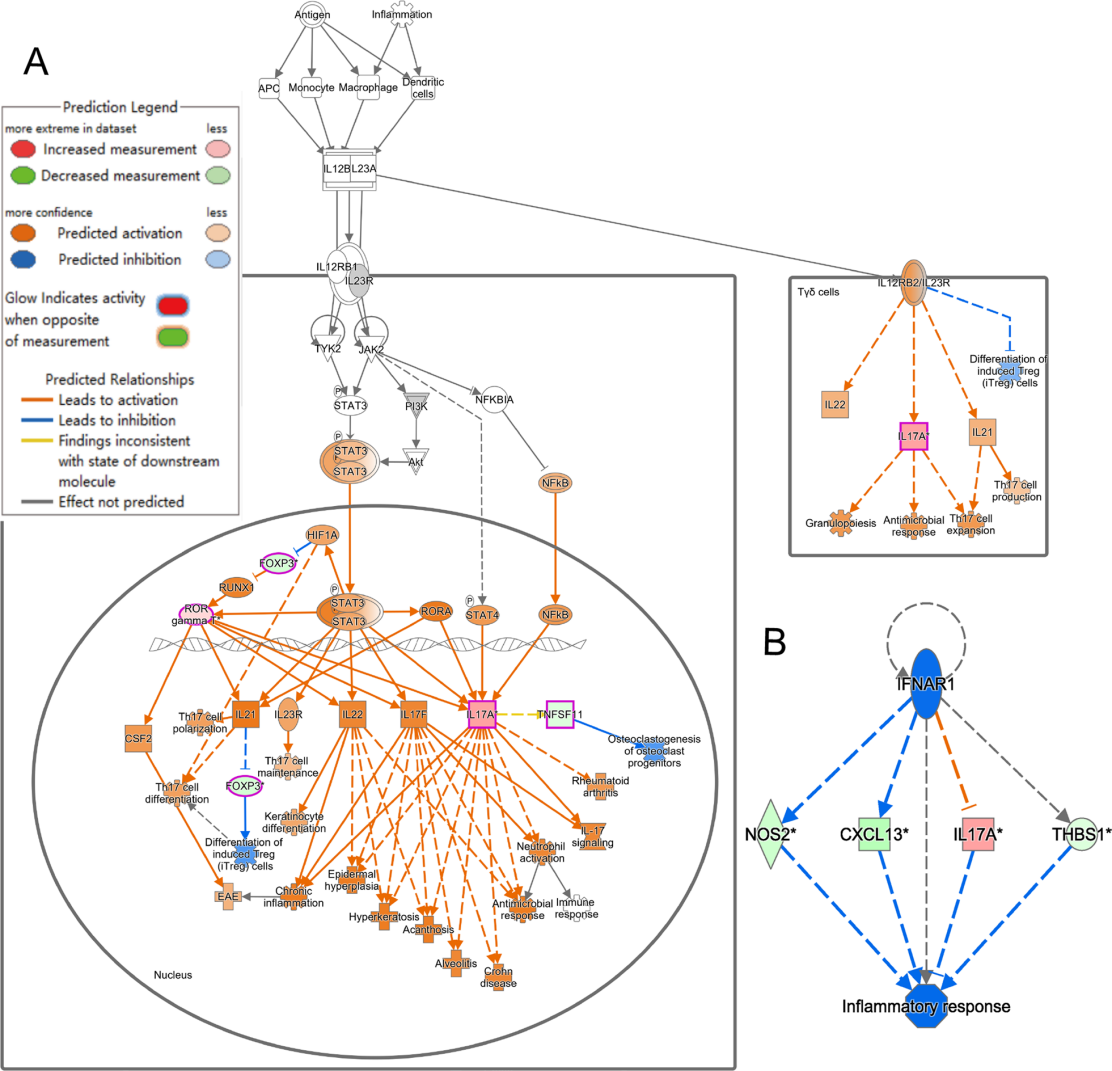
**A** The IL-23 signaling pathway mediated by IL-17A was activated in the Diabetes + Acarbose + Infected compared to Diabetes + Infected group. **B** Network of regulator effects analysis of IL-17A performed using IPA. (green: down-regulated; red: up-regulated; blue: predicted to inhibit; orange: predicted to activate)

Additionally, inhibition of the S100 family NF-κB signaling pathway in immune cells reduced the risk of inflammatory responses and cytokine storms (Fig. 7). Furthermore, the activation of the IL-23 signaling pathway, mediated by IL-17A, promoted antibacterial responses, neutrophil activation, and pneumonia (Fig. 8A). The upstream regulatory factor and regulatory factor effect analysis revealed that upregulated IL-17A may be capable of inhibiting inflammatory responses (Fig. 8B). The functions of immune system activity and inflammation regulated by IL-17A may play a significant role in the mechanism by which acarbose reduces the severity of *P. aeruginosa* infection in T2DM mice. Finally, the qPCR results confirmed that IL-17A expression changes were consistent with the RNA-seq results (Additional file 1: Fig. S5B).

### Acarbose reduces *P. aeruginosa* infection in Ctrl mouse lungs

**Fig. 9:**
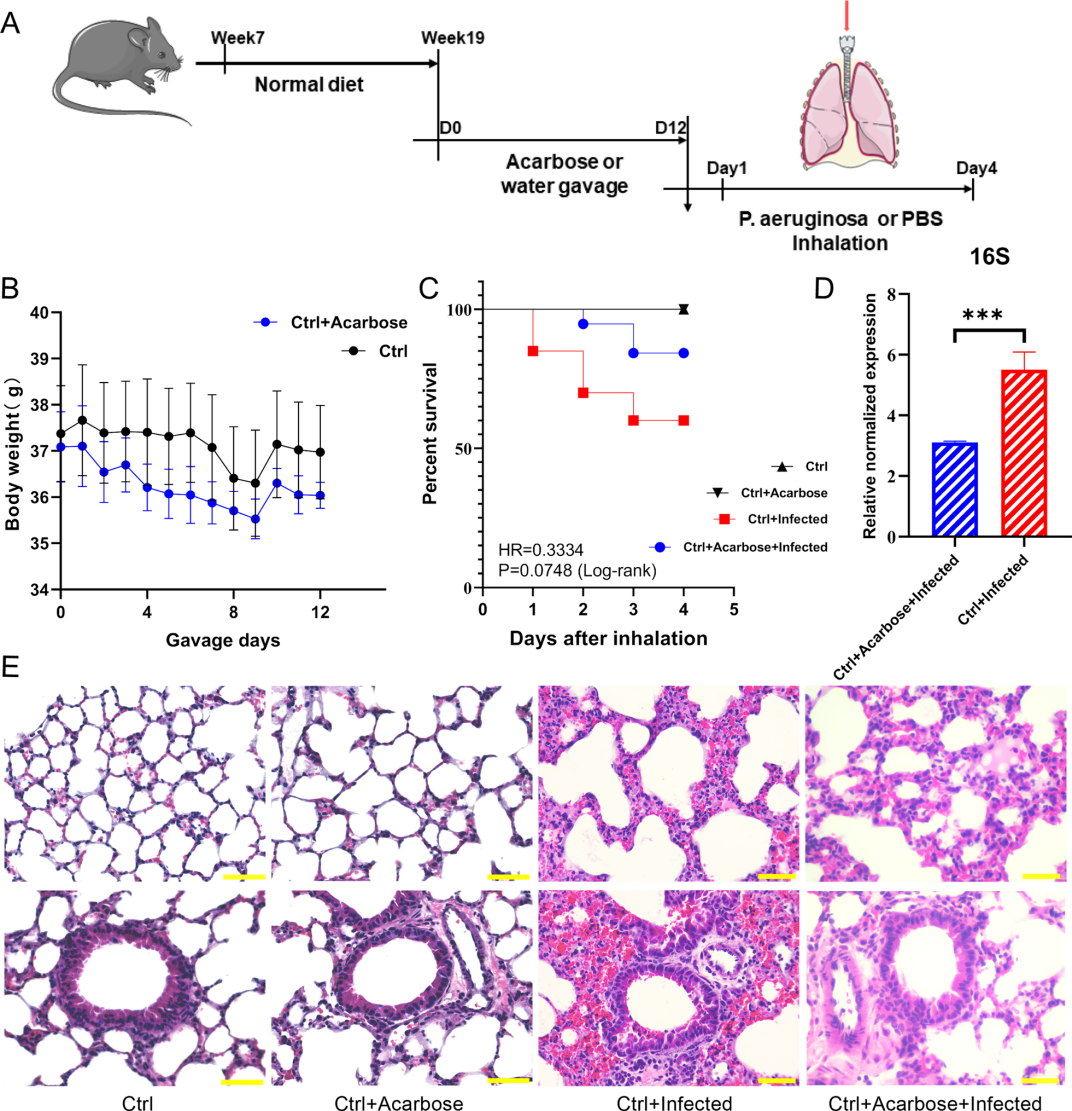
**A** Schematic representation of the experimental design of acarbose gavage and *P. aeruginosa* infection in the control mouse model. **B** Body weights during acarbose treatment in the Ctrl and Ctrl + Acarbose groups (n = 20). **C** Survival curve of the Ctrl, Ctrl + Acarbose, Ctrl + Infected, and Ctrl + Acarbose + Infected groups four days after inhalation. **D** The expression of *P. aeruginosa* in the lung tissue of mice in the Ctrl + Infected and Ctrl + Acarbose + Infected groups (n = 5, ***P < 0.005). **E** H&E staining of lung tissue sections from mice in the Ctrl, Ctrl + Acarbose, Ctrl + Infected, and Ctrl + Acarbose + Infected groups (scale bar = 50 µm)

Acarbose is primarily used to treat diabetes in clinical practice. However, its potential effect on *P. aeruginosa* RTI in nondiabetic individuals has not been thoroughly investigated. In this study, nondiabetic mice were exposed to *P. aeruginosa* tracheal inhalation and treated with acarbose to investigate its effects and underlying mechanisms (Fig. 9A).

The twelve-day acarbose treatment did not significantly affect the body weight of nondiabetic mice (n = 20) (Fig. 9B). Both the Ctrl + Acarbose + Infected and Ctrl + Infected groups experienced weight loss due to *P. aeruginosa* infection, but the degree of weight loss in the Ctrl + Acarbose + Infected group was relatively mild (Additional file 1: Fig. S4E, F).

The four-day survival rate of the Ctrl + Acarbose + Infected group was higher at 84.21% compared to 60% in the Ctrl + Infected group. Furthermore, the HR (Ctrl + Acarbose + Infected VS Ctrl + Infected, 0.3334) showed that acarbose treatment could reduce the risk of death caused by *P. aeruginosa* infection in nondiabetic mice, indicating that acarbose application in nondiabetic individuals also has special significance (Fig. 9C).

H&E-stained lung tissue sections from the Ctrl + Acarbose + Infected and Ctrl + Infected groups exposed to *P. aeruginosa* inhalation showed significant interstitial hemorrhage, lung tissue damage, and disordered airway cell arrangement, with acarbose treatment having a relatively minor effect on the pathological staining results (Fig. 9E). However, the qPCR results of the PAO_1_ 16S primer showed a significant reduction in *P. aeruginosa* expression in the lungs of mice in the Ctrl + Acarbose + Infected group after acarbose treatment, indicating a substantial anti-infective effect (Fig. 9D).

### Acarbose reduces *P. aeruginosa* infection in Ctrl mouse lungs

**Fig. 10:**
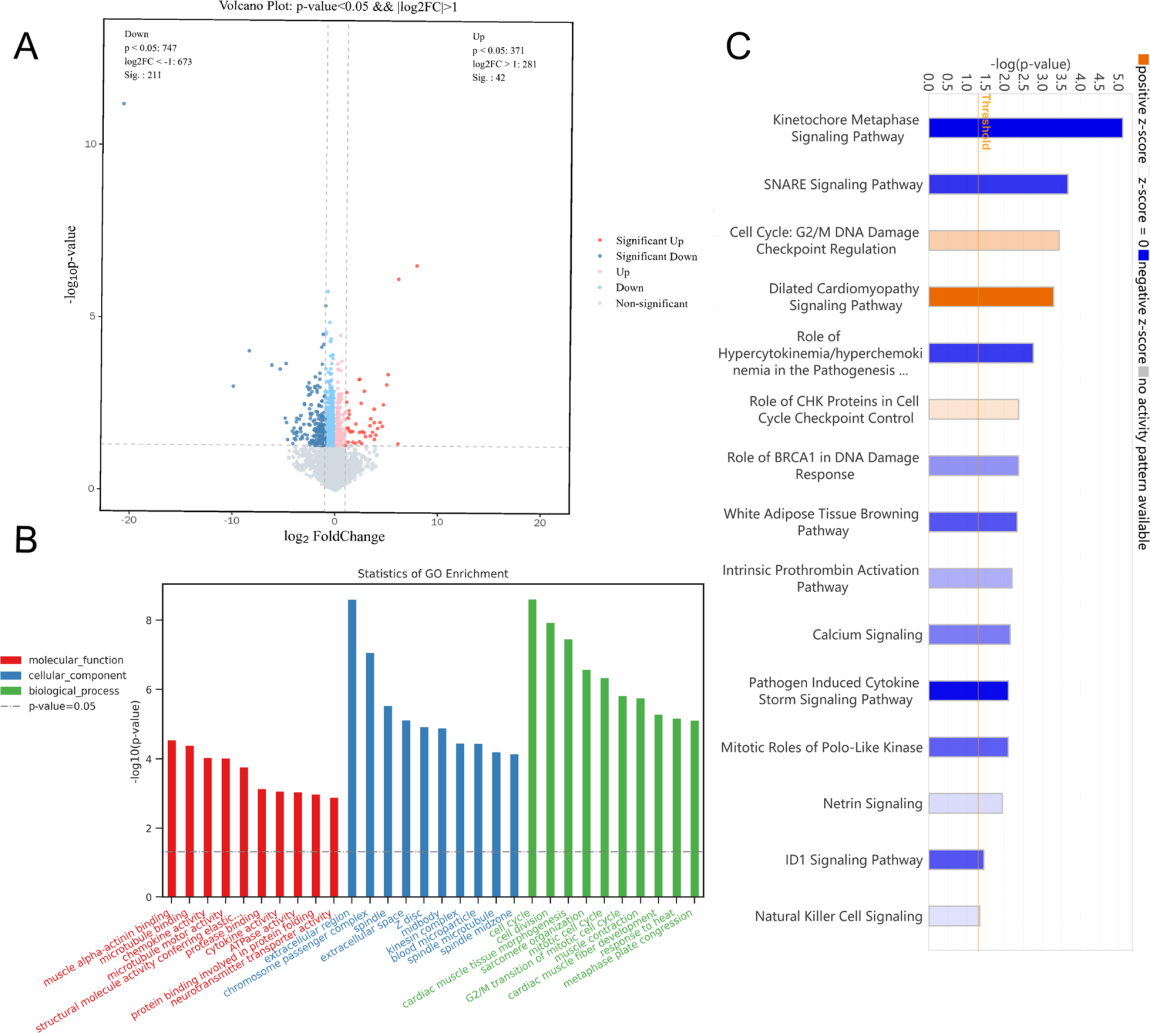
**A** Volcano plot of DEGs between the Ctrl + Infected and Ctrl + Acarbose + Infected groups (Q < 0.05; |log(fold change)|> 1). **B** Bubble chart from GO functional enrichment of the top 10 DEGs between Ctrl + Infected and Ctrl + Acarbose + Infected groups. **C** Bar chart created by using IPA software to analyse the canonical pathways of DEGs between the Ctrl + Infected and Ctrl + Acarbose + Infected groups (P < 0.05; |Z score|≥ 0)

Compared to the Ctrl + Infected group, transcriptomic analysis of the Ctrl + Acarbose + Infected group revealed 42 upregulated DEGs and 211 downregulated DEGs (Fig. 10A). GO functional analysis of DEGs between the two groups demonstrated significant enrichment in functions related to the cell cycle, microtubules, and cytokines (Fig. 10B).

**Fig. 11:**
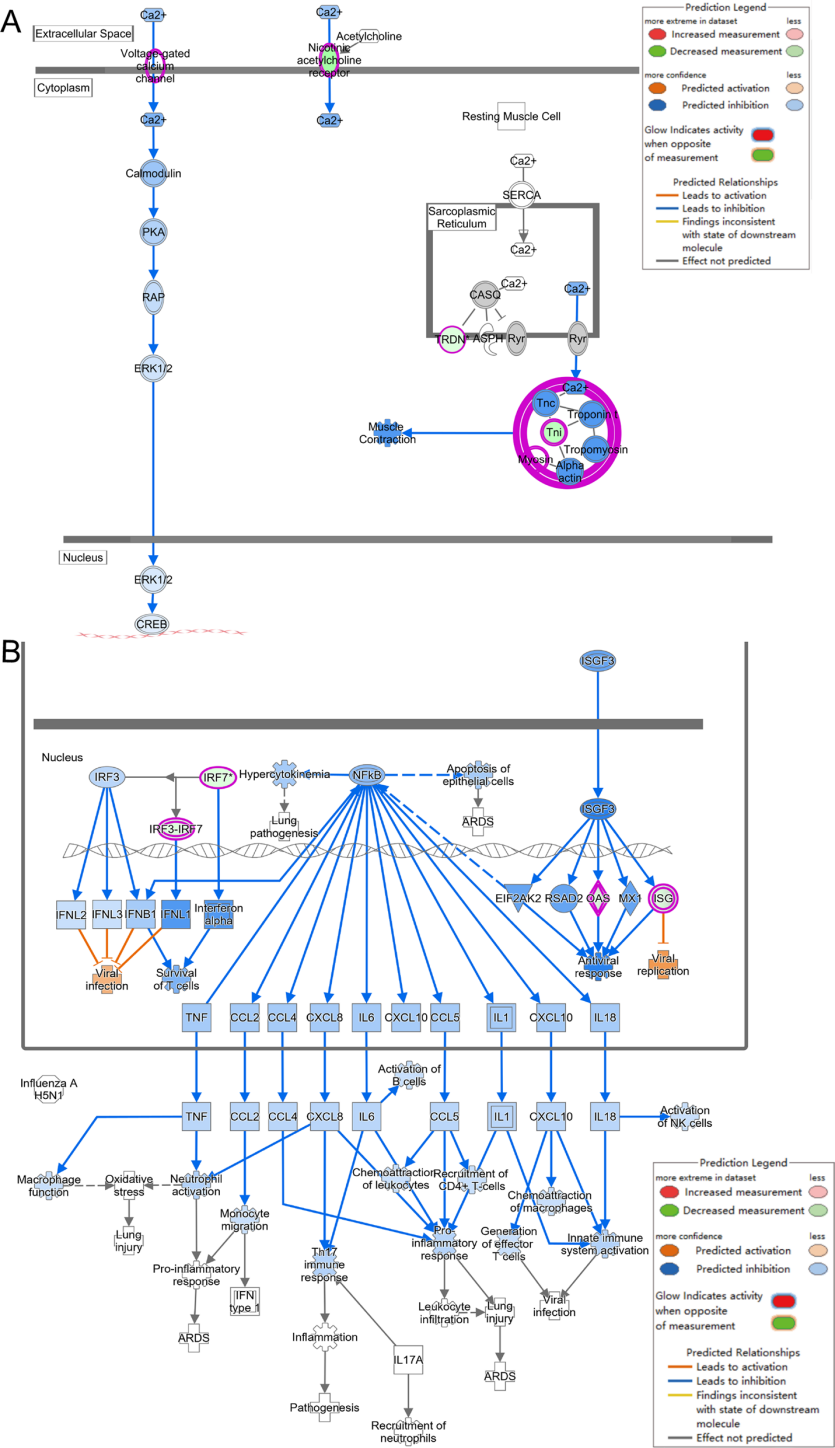
**A** The calcium signaling pathway and **B** NF-κB signaling pathway were activated in the Ctrl + Acarbose + Infected compared to the Ctrl + Infected group (green: down-regulated; red: up-regulated; blue: predicted to inhibit; orange: predicted to activate)

Furthermore, Canonical pathway analysis of the above DEGs by IPA software indicated that the pathogen-induced cytokine storm signaling pathway (Z score = − 3.051), calcium signaling pathway (Z score = − 1.633), and natural killer cell signaling pathway (Z score = − 0.378) were suppressed, in addition to changes in pathways related to the cell cycle (Fig. 10C). The downregulation of cell membrane calcium ion channels and the inhibition of endoplasmic reticulum calcium ion release suggest the possible suppression of the calcium ion signaling pathway, leading to a decrease in intracellular calcium ion concentration (Fig. 11A). Moreover, the NF-κB pathway, which regulates the inflammatory response, was suppressed, providing a possible mechanism for the effect of acarbose on the inflammatory response of non-diabetic mice with *P. aeruginosa* RTI (Fig. 11B).

## Discussion

In recent years, there has been increasing recognition that individuals with T2DM suffer from exacerbated *P. aeruginosa* infections [18, 52]. Nevertheless, current studies still primarily focus on statistical analysis of clinical data. There is a need to further explore the underlying molecular mechanisms. At the same time, recent studies have reported on the alleviating effects of acarbose on RTIs and inflammatory responses [33, 34, 53, 54]. As an effective postprandial blood glucose reducer, acarbose was widely applied in the clinical treatment of T2DM [39, 55, 56]. However, compared to other clinically used antidiabetic drugs [57, 58], there is currently relatively little research on the impact and mechanism of acarbose on RTIs in T2DM individuals.

The results of our study indicated, using an animal model, that T2DM can exacerbate *P. aeruginosa* RTIs and increase mortality risk. This is consistent with previous clinical data and experimental results [22–26]. Additionally, the study found that acarbose treatment can alleviate the severity of *P. aeruginosa* RTI in T2DM mice. It can also reduce their mortality, and surprisingly, exhibit a certain degree of anti-infective effect in non-diabetic mice with *P. aeruginosa* RTI.

Our study discovered that the NF-κB signaling pathway was significantly activated in T2DM mice after *P. aeruginosa* infection, compared to that in non-diabetic mice. There is evidence that the NF-κB signaling pathway plays a crucial role in *P. aeruginosa* survival within cells, which is triggered by its initial acute infection. Long-term activation of the NF-κB signaling pathway may contribute to chronic persistent inflammation and long-term colonization of *P. aeruginosa* [59–61]. Therefore, this activation is significantly correlated with the less effective immune response to *P. aeruginosa* infection in T2DM individuals, leading to persistent infection and inflammation.

Similar to the NF-κB signal, TREM-1 signal activation amplifies the inflammatory response caused by bacterial infection. Blocking TREM-1 signal transduction can prolong the survival of *P. aeruginosa* infected mice [62]. Thus, the Diabetes + Infected group showed significant activation of the TREM-1 signaling pathway compared with the Ctrl + Infected group, likely exacerbating the infection.

In conclusion, the activation of the TREM-1 signaling pathway, along with the activation of the NF-κB signaling pathway and pathogen-induced cytokine storm signaling pathway, collectively mediates the exacerbation of *P. aeruginosa* RTI in T2DM mice. Our study effectively explores the impact of T2DM on *P. aeruginosa* RTI and its possible mechanisms, providing potential therapeutic targets for controlling this complex disease condition.

Moreover, in our study, the inhibition of the HIF-1α signaling pathway was found to be associated with the inhibitory and prognostic improvement effects of acarbose on *P. aeruginosa* RTI in T2DM mice. HIF-1α is an effective regulator of innate immunity that can inhibit the innate immune response of airway epithelial cells and promote bacterial infection, while *P. aeruginosa* secreted factors significantly inhibit its function [63–66]. However, another study has shown that HIF-1 knockout in Caenorhabditis elegans can exacerbate *P. aeruginosa* pathogenesis [67]. In future studies, we will further determine the role of the HIF-1α signaling pathway in *P. aeruginosa* infection and explore its mechanism.

It is noteworthy that our study found that oral acarbose had a therapeutic effect on *P. aeruginosa* RTI in both diabetic and non-diabetic mice. In both groups, the NF-κB signaling pathway showed significant inhibition after acarbose treatment. The inflammatory response mediated by NF-κB is the primary pathway induced by *P. aeruginosa* infection [60, 61, 68]. As a result, acarbose may be able to alleviate *P. aeruginosa* RTI severity in mice by inhibiting the NF-κB signaling pathway, independent of blood glucose levels.

Furthermore, following acarbose treatment, the calcium signaling pathway was inhibited in non-diabetic mice with *P. aeruginosa* RTI, which is different from T2DM mice. Previous studies suggest that *P. aeruginosa* activates the calcium ion signaling pathway, activating the inflammatory signal and causing excessive airway inflammation [69, 70]. Consequently, calcium ion signaling pathway inhibition in this section's experimental results may be associated with acarbose's ability to alleviate *P. aeruginosa* infection in non-diabetic mice.

In previous studies, the mechanism of action of antidiabetic drugs against infection has mainly been investigated based on their hypoglycemic effects [34, 53]. However, our study demonstrated that acarbose has an inhibitory effect on *P. aeruginosa* RTI not only in T2DM mice but also in non-diabetic mice, indicating an anti-infective function independent of blood glucose and lung glucose levels. Moreover, we observed that the molecular mechanisms of acarbose against *P. aeruginosa* RTI in T2DM and non-diabetic mice are not completely identical. This may be because acarbose has some potential to help fight RTIs, as previously discovered in its potential use for COVID-19 patients. Our future studies will focus on modulating the activation or inhibition of the aforementioned signaling pathways to explore their specific causal effects on these diseases.

In summary, our study found that (1) the exacerbation of *P. aeruginosa* RTI in T2DM is attributed to the activation of the NF-κB and TREM-1 signaling pathways, (2) the alleviation of *P. aeruginosa* RTI by acarbose in T2DM individuals is related to the inhibition of the NF-κB and HIF-1α signaling pathways, and (3) this alleviation in non-diabetic individuals is associated with inhibiting the NF-κB and calcium signaling pathways. Our study identifies the preventive and therapeutic effects of acarbose on respiratory tract *P. aeruginosa* infection in T2DM and non-diabetic individuals and explores its potential mechanisms, providing new support for its clinical application as an anti-infective or adjuvant medication.

### Supplementary Information

**Additional file 1: Figure S1.** (A) Macroscopic inflammatory damage in the Ctrl + Infected group mice lungs at day 4 post-inhalation (scale bars = 1 cm). (B) Bacterial loads (CFU) in mice lungs homogenates were determined by serial dilution on Luria broth agar (Sigma-Aldrich, UK) at day 4 post-infection (n = 3, ***P < 0.005). (C) Changes in mice body temperature in the four days after inhalation. (D) Changes in qPCR detection results in mouse lung tissue after inhalation for four days. (n = 5, ***P < 0.005). **Figure S2.** Growth curve of *P. aeruginosa* PAO1 (Control VS Acarbose 2 mg/ml). Experiments performed in triplicate were repeated at least four times. **Figure S3.** Oral glucose tolerance test (OGTT) in Ctrl and HFD mice. Time course of OGTT after orally administered glucose at a dose of 2 g/kg (A) and area under curve (AUC) (B). (n = 5, ***P < 0.005). **Figure S4.** (A ~ B) Body weights and their percentage of original body weights of Ctrl, Diabetes, Ctrl + Infected, and Diabetes + Infected groups in the four days after inhalation (n = 10, ***P < 0.005, * P < 0.1). (C ~ D) Body weights and their percentage of original body weights of Diabetes and Diabetes + Acarbose, Diabetes + Infected, and Diabetes + Acarbose + Infected groups in the four days after inhalation (n = 10, ***P < 0.005, * P < 0.1). (E ~ F) Body weights and their percentage of original body weights of Ctrl, Ctrl + Acarbose, Ctrl + Infected, and Ctrl + Acarbose + Infected groups in the four days after inhalation (n = 10, ***P < 0.005, * P < 0.1). **Figure S5.** (A) Using qPCR verification the DEGs in RNA-seq result (Diabetes + Infected VS Ctrl + Infected). (B) Using qPCR verification the DEGs in RNA-seq result (Diabetes + Acarbose + Infected VS Diabetes + Infected).

**Additional file 2: Table S1.** The sequences of primers for qPCR. **Table S2.** Top regulated genes in the Diabetes + Infected VS Ctrl + Infected. **Table S3.** Top regulated genes in the Diabetes + Acarbose + Infected VS Diabetes + Infected. **Table S4.** Top regulated genes in the Ctrl + Acarbose + Infected VS Ctrl + Infected.

## Data Availability

All raw sequencing data and any other materials that support the findings of this study are available from the corresponding author upon reasonable request and needs to abide by approved ethical and legal policies. Processed data is also presented in Additional file 2: Tables S2, S3 and S4.

## Abbreviations

T2DM: Type 2 diabetes mellitus
RTI: Respiratory tract infection
COVID-19: Coronavirus disease 2019
NF-κB: Nuclear factor kappa-B
TREM-1: Triggering receptor expressed on myeloid cells-1
SPF: Specific pathogen-free
PBS: Phosphate buffer saline
HFD: High-fat diet
HIF-1α: Hypoxia-inducible factor-1α
CFU: Colony forming unit
H&E: Hematoxylin and eosin
DEGs: Differentially expressed genes
GO: Gene Ontology
KEGG: Kyoto Encyclopedia of Genes and Genomes
qPCR: Quantitative polymerase chain reaction
IPA: Ingenuity pathway analysis
SEM: Standard error of the mean
MF: Molecular function
CC: Cellular component
BP: Biological process
TNF: Tumor necrosis factor
IL: Interleukin
NOS2: Nitric oxide synthase 2
