# Supplementary material for: Acarbose reduces Pseudomonas aeruginosa respiratory tract infection in type 2 diabetic mice
Source: Respir Res. 2023 Dec 14;24:312. doi: 10.1186/s12931-023-02619-8 (PMC10722695; doi:10.1186/s12931-023-02619-8)
Supplement: Supplementary file 1 — Additional file 1: Figure S1. (A) Macroscopic inflammatory damage in the Ctrl + Infected group mice lungs at day 4 post-inhalation (scale bars = 1 cm). (B) Bacterial loads (CFU) in mice lungs homogenates were determined by serial dilution on Luria broth agar (Sigma-Aldrich, UK) at day 4 post-infection (n = 3, ***P < 0.005). (C) Changes in mice body temperature in the four days after inhalation. (D) Changes in qPCR detection results in mouse lung tissue after inhalation for four days. (n = 5, ***P < 0.005). Figure S2. Growth curve of P. aeruginosa PAO1 (Control VS Acarbose 2 mg/ml). Experiments performed in triplicate were repeated at least four times. Figure S3. Oral glucose tolerance test (OGTT) in Ctrl and HFD mice. Time course of OGTT after orally administered glucose at a dose of 2 g/kg (A) and area under curve (AUC) (B). (n = 5, ***P < 0.005). Figure S4. (A ~ B) Body weights and their percentage of original body weights of Ctrl, Diabetes, Ctrl + Infected, and Diabetes + Infected groups in the four days after inhalation (n = 10, ***P < 0.005, * P < 0.1). (C ~ D) Body weights and their percentage of original body weights of Diabetes and Diabetes + Acarbose, Diabetes + Infected, and Diabetes + Acarbose + Infected groups in the four days after inhalation (n = 10, ***P < 0.005, * P < 0.1). (E ~ F) Body weights and their percentage of original body weights of Ctrl, Ctrl + Acarbose, Ctrl + Infected, and Ctrl + Acarbose + Infected groups in the four days after inhalation (n = 10, ***P < 0.005, * P < 0.1). Figure S5. (A) Using qPCR verification the DEGs in RNA-seq result (Diabetes + Infected VS Ctrl + Infected). (B) Using qPCR verification the DEGs in RNA-seq result (Diabetes + Acarbose + Infected VS Diabetes + Infected). [file 12931_2023_2619_MOESM1_ESM.docx]

**Additional Figures**

**Figure S1**

**
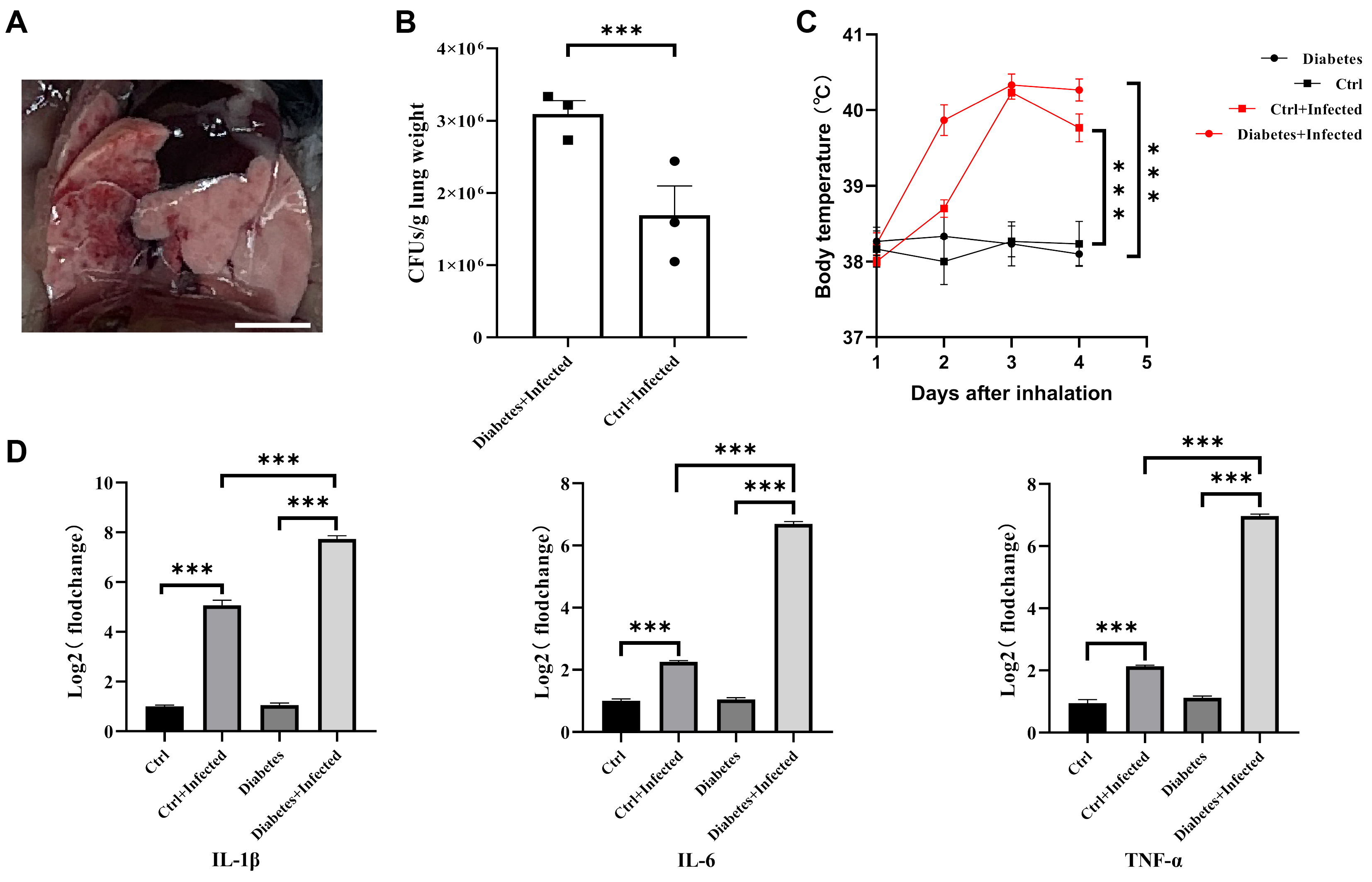
**

Figure S1. (A) Macroscopic inflammatory damage in the Ctrl+Infected group mice lungs at day 4 post-inhalation (scale bars=1 cm). (B) Bacterial loads (CFU) in mice lungs homogenates were determined by serial dilution on Luria broth agar (Sigma-Aldrich, UK) at day 4 post-infection (n=3, ***P<0.005). (C) Changes in mice body temperature in the four days after inhalation. (D) Changes in qPCR detection results in mouse lung tissue after inhalation for four days. (n=5, ***P<0.005)

**Figure S2**

**
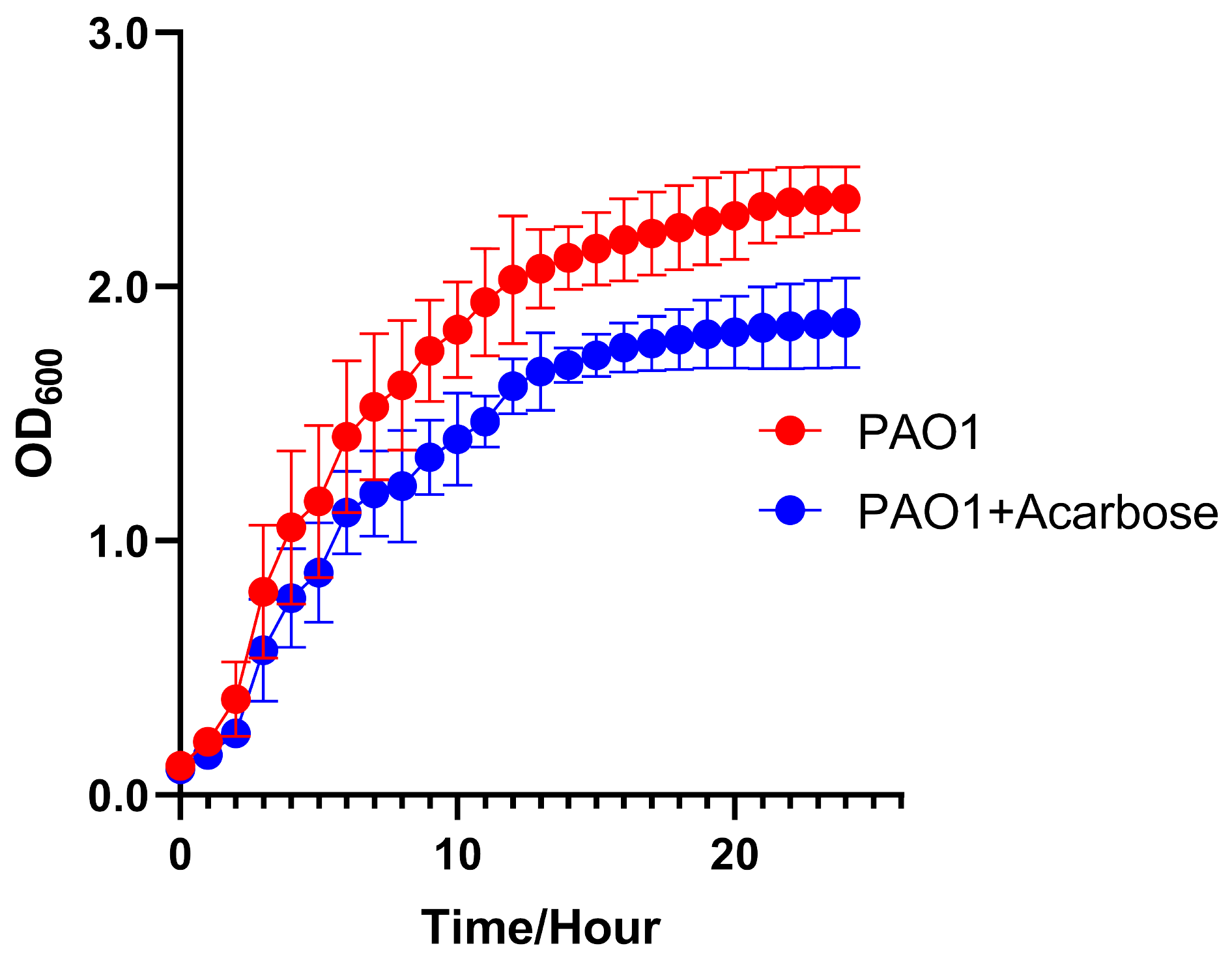
**

Figure S2. Growth curve of *P. aeruginosa* PAO1 (Control VS Acarbose 2 mg/ml). Experiments performed in triplicate were repeated at least four times.

**Figure S3**


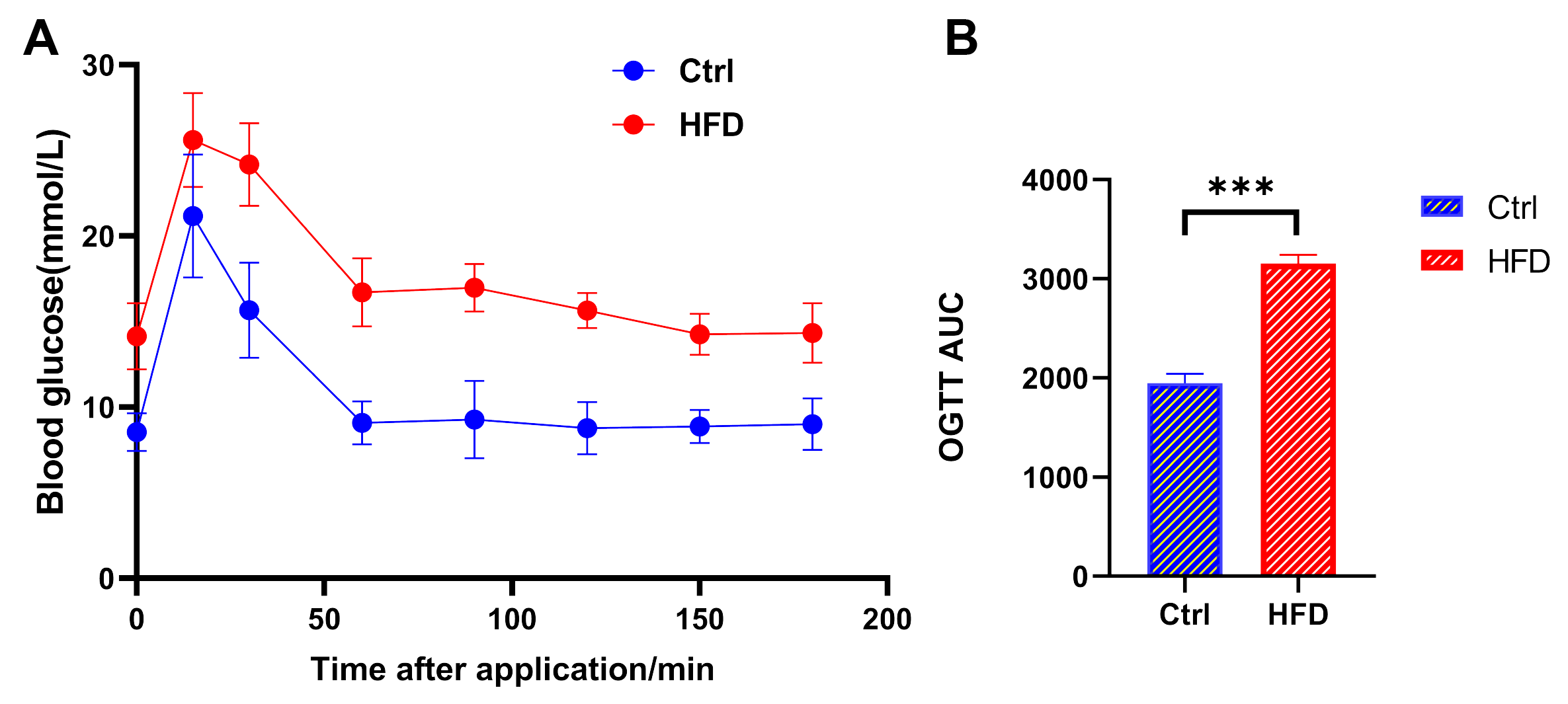


Figure S3. Oral glucose tolerance test (OGTT) in Ctrl and HFD mice. Time course of OGTT after orally administered glucose at a dose of 2 g/kg (A) and area under curve (AUC) (B). (n=5, ***P<0.005)

**Figure S4**

**
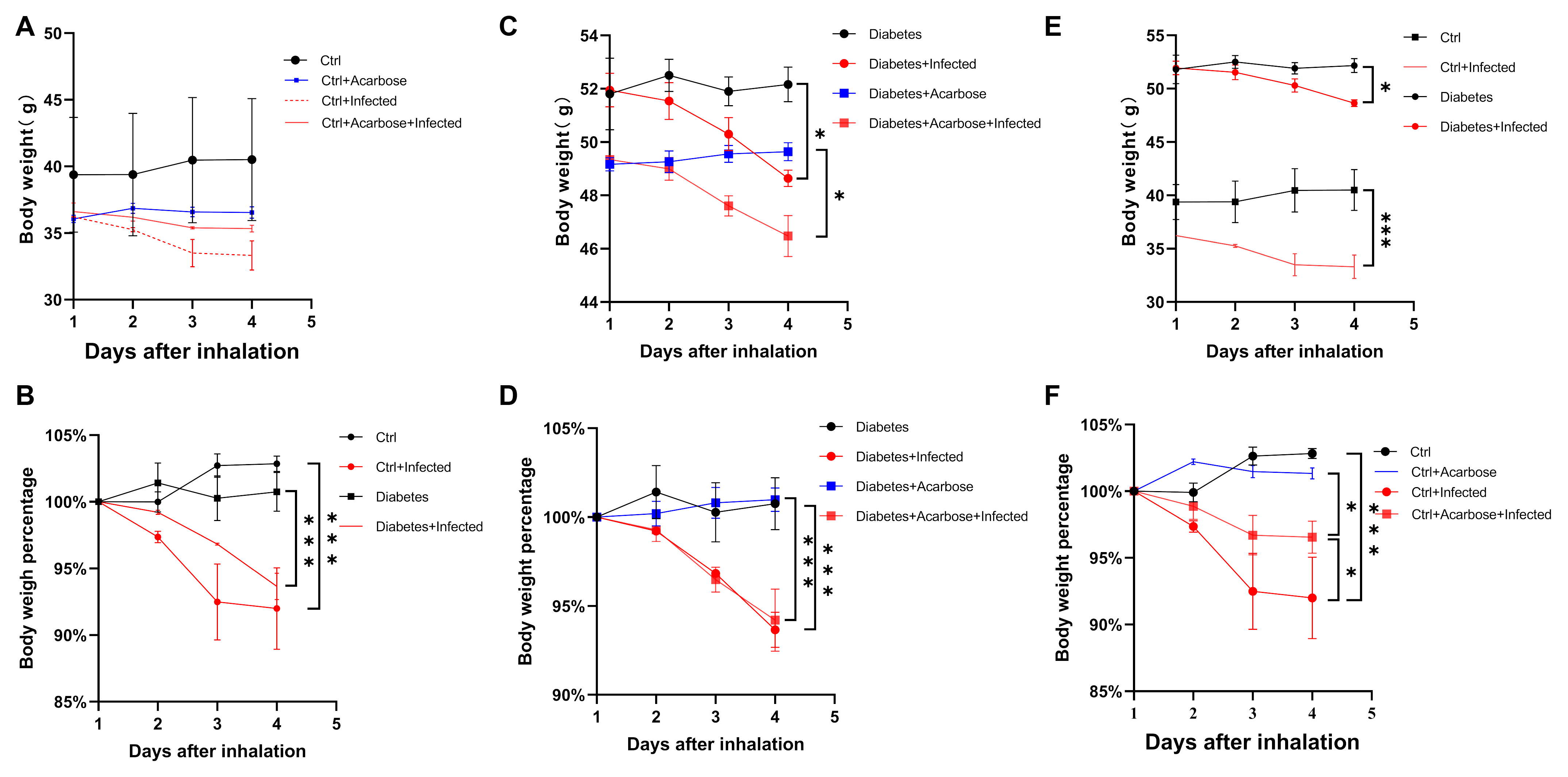
**

Figure S4. (A~B) Body weights and their percentage of original body weights of Ctrl, Diabetes, Ctrl+Infected, and Diabetes+Infected groups in the four days after inhalation (n=10, ***P<0.005, * P<0.1). (C~D) Body weights and their percentage of original body weights of Diabetes and Diabetes+Acarbose, Diabetes+Infected, and Diabetes+Acarbose+Infected groups in the four days after inhalation (n=10, ***P<0.005, * P<0.1). (E~F) Body weights and their percentage of original body weights of Ctrl, Ctrl+Acarbose, Ctrl+Infected, and Ctrl+Acarbose+Infected groups in the four days after inhalation (n=10, ***P<0.005, * P<0.1).

**Figure S5**

**
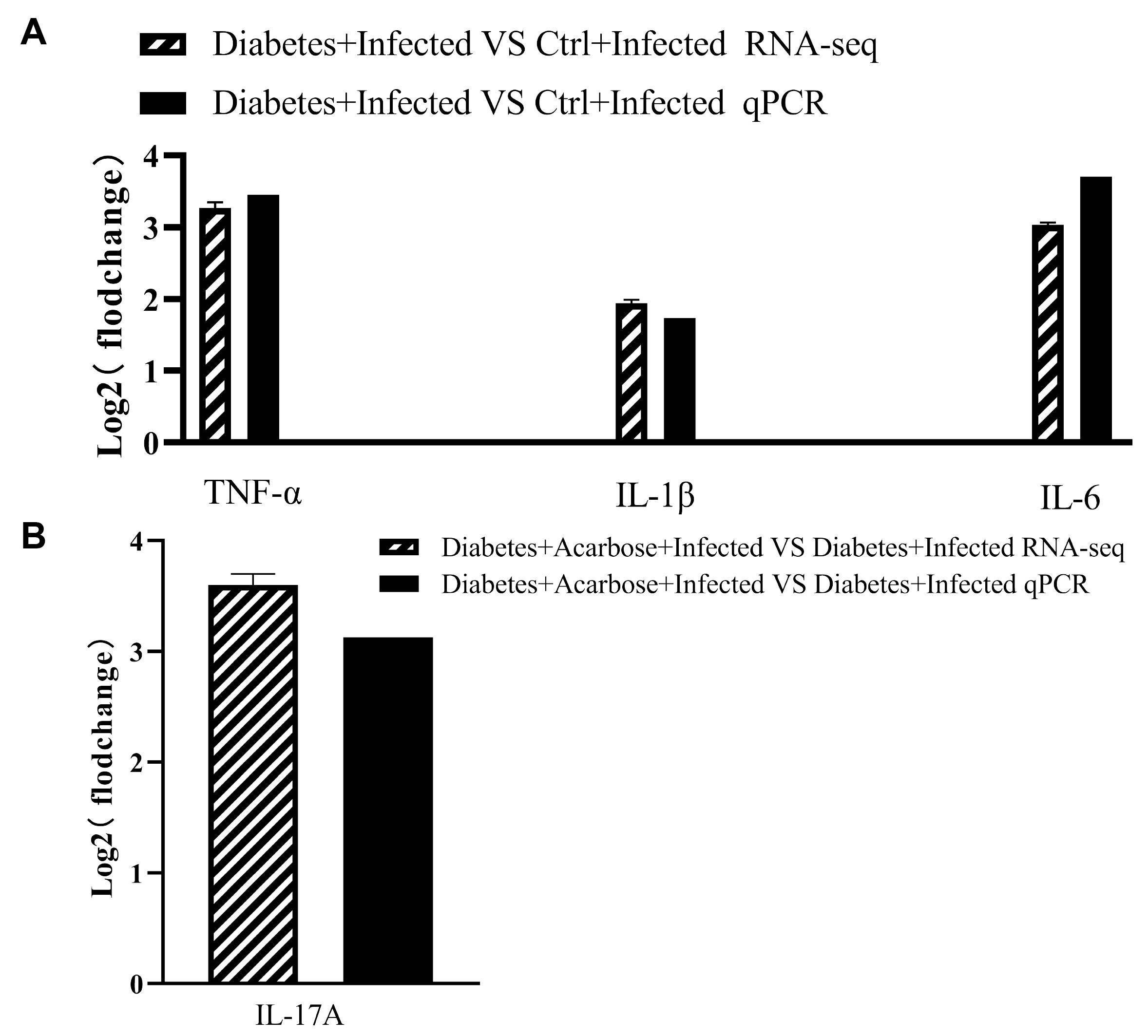
**

Figure S5. (A)Using qPCR verification the DEGs in RNA-seq result (Diabetes+Infected VS Ctrl+Infected). (B)Using qPCR verification the DEGs in RNA-seq result (Diabetes+Acarbose+Infected VS Diabetes+Infected).
