# Supplementary material for: Acarbose reduces Pseudomonas aeruginosa respiratory tract infection in type 2 diabetic mice
Source: Respir Res. 2023 Dec 14;24:312. doi: 10.1186/s12931-023-02619-8 (PMC10722695; doi:10.1186/s12931-023-02619-8)
Supplement: Supplementary file 2 — Additional file 2: Table S1. The sequences of primers for qPCR. Table S2. Top regulated genes in the Diabetes + Infected VS Ctrl + Infected. Table S3. Top regulated genes in the Diabetes + Acarbose + Infected VS Diabetes + Infected. Table S4. Top regulated genes in the Ctrl + Acarbose + Infected VS Ctrl + Infected. [file 12931_2023_2619_MOESM2_ESM.docx]

**Table S1. The sequences of primers for qPCR**

|  | F | R |
| --- | --- | --- |
| PAO_1_ 16SRNA | CCCAACATCTCACGACACGA | ACGCGAAGAACCTTACCTGG |
| M IL-1β | GAAATGCCACCTTTTGACAGTG | TGGATGCTCTCATCAGGACAG |
| M IL-6 | TCTATACCACTTCACAAGTCGGA | GAATTGCCATTGCACAACTCTTT |
| M IL-10 | CTTACTGACTGGCATGAGGATCA | GCAGCTCTAGGAGCATGTGG |
| M TNF-α | CCCTCACACTCAGATCATCTTC | GCTACGACGTGGGCTACAG |
| M IL-17 | TCAGCGTGTCCAAACACTGAG | CGCCAAGGGAGTTAAAGACTT |
| M 18S | GTAACCCGTTGAACCCCATT | CCATCCAATCGGTAGTAGCG |

**Table S2. Top regulated genes in the Diabetes+Infected VS Ctrl+Infected**

| **Gene Name** | **Gene ID** | **log2FoldChange** | **p-value** | **GO ID** | **Gene Description** | |
| --- | --- | --- | --- | --- | --- | --- |
| Top 50 up-regulated genes in Diabetes+Infected VS Ctrl+Infected | | | | | | |
| Prok2 | 50501 | 9.413098 | 1.02E-06 | GO:0000187,GO:0001525,GO:0001664,GO:0001935 | prokineticin 2 |  |
| Saa2 | 20209 | 6.207887 | 0.035717 | GO:0001664,GO:0005515,GO:0005576,GO:0005881 | serum amyloid A 2 |  |
| Csf3 | 12985 | 5.84045 | 0.046211 | GO:0005125,GO:0005130,GO:0005576,GO:0005615 | colony stimulating factor 3 (granulocyte) |  |
| Ngp | 18054 | 5.748931 | 0.000288 | GO:0004869,GO:0005576,GO:0005615,GO:0006952 | neutrophilic granule protein |  |
| Gm6551 | 625060 | 5.625752 | 0.044497 |  | predicted gene 6551 |  |
| Stfa3 | 20863 | 5.530405 | 0.047856 | GO:0004869,GO:0005829 | stefin A3 |  |
| Saa1 | 20208 | 5.362392 | 0.005105 | GO:0001664,GO:0005515,GO:0005576,GO:0005615 | serum amyloid A 1 |  |
| Gm4832 | 225058 | 5.147474 | 0.00796 |  | predicted gene 4832 |  |
| Ms4a3 | 170813 | 5.049368 | 0.012147 | GO:0005515,GO:0005737,GO:0016020,GO:0016021,GO:0051726 | membrane-spanning 4-domains,  subfamily A, member 3 |  |
| Olfm4 | 380924 | 4.505235 | 0.000819 | GO:0005198,GO:0005515,GO:0005576,GO:0005615 | olfactomedin 4 |  |
| Gm34362 | 102637593 | 4.426336 | 0.030213 |  |  |  |
| Itgb2l | 16415 | 4.366418 | 0.004212 | GO:0001540,GO:0005178,GO:0005886,GO:0005925 | integrin beta 2-like |  |
| Cldn13 | 57255 | 4.1176 | 0.028049 | GO:0005198,GO:0005886,GO:0005923,GO:0007155 | claudin 13 |  |
| Ccl3 | 20302 | 4.064296 | 0.00194 | GO:0002548,GO:0005125,GO:0005515,GO:0005576 | chemokine (C-C motif) ligand 3 |  |
| Saa3 | 20210 | 4.050517 | 0.025721 | GO:0005576,GO:0005615,GO:0006953,GO:0007252 | serum amyloid A 3 |  |
| Stfa2 | 20862 | 4.007204 | 0.043523 | GO:0004866,GO:0004869,GO:0005737,GO:0005829 | stefin A2 |  |
| Mymk | 66139 | 3.973674 | 0.010384 | GO:0005515,GO:0005794,GO:0005886,GO:0005887 | myomaker,  myoblast fusion factor |  |
| Wee2 | 381759 | 3.814003 | 0.035407 | GO:0000166,GO:0000278,GO:0000287,GO:0004672, | WEE1 homolog 2  (S. pombe) |  |
| LOC100039029 | 100039029 | 3.78559 | 0.034215 |  |  |  |
| Atp8b3 | 67331 | 3.778528 | 0.002581 | GO:0000166,GO:0000287,GO:0001669,GO:0005524 | ATPase, class I, type 8B, member 3 |  |
| 6030468B19Rik | 77727 | 3.771588 | 0.034984 | GO:0002250,GO:0002313,GO:0002376,GO:0004888 | RIKEN cDNA  6030468B19 gene |  |
| Kng1 | 16644 | 3.761515 | 0.006587 | GO:0004866,GO:0004869,GO:0005102,GO:0005576 | kininogen 1 |  |
| Camp | 12796 | 3.748328 | 0.01885 | GO:0001530,GO:0001878,GO:0001934,GO:0002227 | cathelicidin antimicrobial peptide |  |
| Olfr1396 | 258334 | 3.631574 | 0.01172 | GO:0004984,GO:0007186,GO:0007608,GO:0016021 | olfactory receptor 1396 |  |
| Enthd1 | 383075 | 3.588867 | 0.013139 | GO:0005543,GO:0005768,GO:0005886,GO:0006897 | ENTH domain containing 1 |  |
| Bcl2l15 | 229672 | 3.550182 | 0.036334 | GO:0005634,GO:0005829,GO:0042981 | BCLl2-like 15 |  |
| Fcnb | 14134 | 3.544729 | 0.000333 | GO:0001664,GO:0001867,GO:0002376,GO:0002752 | ficolin B |  |
| Asxl3 | 211961 | 3.470822 | 0.046867 | GO:0003677,GO:0003682,GO:0005634,GO:0006351,GO:0006355,GO:0009887 | additional sex combs like 3,  transcriptional regulator |  |
| Ifitm6 | 213002 | 3.437873 | 0.001056 | GO:0005886,GO:0034341,GO:0035455,GO:0035456 | interferon induced transmembrane protein 6 |  |
| Gstt4 | 75886 | 3.419824 | 0.016017 | GO:0004364,GO:0005737,GO:0006749,GO:0016740 | glutathione S-transferase, theta 4 |  |
| Arg1 | 11846 | 3.347483 | 0.038828 | GO:0000050,GO:0001938,GO:0002250,GO:0002376 | arginase, liver |  |
| Ear6 | 93719 | 3.318969 | 2.72E-06 | GO:0002227,GO:0004540,GO:0005615,GO:0006935 | eosinophil-associated, ribonuclease A family, member 6 |  |
| Btnl10 | 192194 | 3.270852 | 0.012356 | GO:0001817,GO:0005102,GO:0009897,GO:0016020,GO:0016021,GO:0050852 | butyrophilin-like 10 |  |
| Car1 | 12346 | 3.23128 | 0.020297 | GO:0004064,GO:0004089,GO:0005737,GO:0006730 | carbonic anhydrase 1 |  |
| Cxcl3 | 330122 | 3.167512 | 0.044884 | GO:0005125,GO:0005515,GO:0005576,GO:0005615 | chemokine (C-X-C motif) ligand 3 |  |
| Mmp8 | 17394 | 3.093162 | 0.003486 | GO:0004175,GO:0004222,GO:0004252,GO:0005509 | matrix metallopeptidase 8 |  |
| Ccl4 | 20303 | 3.052009 | 0.001771 | GO:0002548,GO:0005125,GO:0005515,GO:0005576 | chemokine (C-C motif) ligand 4 |  |
| Apol8 | 239552 | 3.037412 | 0.005289 | GO:0008289 | apolipoprotein L 8 |  |
| C1rb | 667277 | 3.036598 | 0.002742 | GO:0002376,GO:0004252,GO:0005509,GO:0005615 | complement component 1, r subcomponent B |  |
| Rhag | 19743 | 3.014137 | 0.012693 | GO:0005886,GO:0005887,GO:0006873,GO:0008519 | Rhesus blood group-associated A glycoprotein |  |
| Ly6g | 546644 | 2.988589 | 0.00634 | GO:0005886,GO:0009897,GO:0016020,GO:0031225 | lymphocyte antigen 6 complex, locus G |  |
| Tspo2 | 70026 | 2.931782 | 0.000145 | GO:0005783,GO:0005886,GO:0015485,GO:0016020 | translocator protein 2 |  |
| Cd5l | 11801 | 2.89779 | 0.005259 | GO:0002376,GO:0004252,GO:0005044,GO:0005576 | CD5 antigen-like |  |
| Ccl2 | 20296 | 2.879137 | 0.03502 | GO:0001525,GO:0001664,GO:0001912,GO:0001938 | chemokine (C-C motif) ligand 2 |  |
| Slc6a3 | 13162 | 2.809024 | 0.024636 | GO:0001504,GO:0002020,GO:0005102,GO:0005326,GO:0005330,GO:0005334,GO:0005515,GO:0005886, | solute carrier family 6 (neurotransmitter transporter, dopamine), member 3 |  |
| Clca1 | 23844 | 2.767557 | 0.004618 | GO:0005229,GO:0005254,GO:0005576,GO:0005887 | chloride channel accessory 1 |  |
| Spic | 20728 | 2.756795 | 0.008311 | GO:0000978,GO:0000981,GO:0001228,GO:0001824 | Spi-C transcription factor (Spi-1/PU.1 related) |  |
| Cpa1 | 109697 | 2.727064 | 0.032584 | GO:0004180,GO:0004181,GO:0005576,GO:0005615 | carboxypeptidase A1, pancreatic |  |
| Fcrlb | 435653 | 2.717219 | 0.022116 | GO:0004888,GO:0005737,GO:0005783,GO:0005887,GO:0007166,GO:0050777 | Fc receptor-like B |  |
| Gm1123 | 382097 | 2.715755 | 0.010931 |  | predicted gene 1123 |  |
| Top 50 down-regulated genes in Diabetes+Infected VS Ctrl+Infected | | | | | | |
| Bmp10 | 12154 | -9.96668 | 0.00099 | GO:0001822,GO:0005125,GO:0005179,GO:0005576 | bone morphogenetic protein 10 |  |
| Gm4779 | 102634296 | -6.21859 | 0.014654 |  | predicted gene 4779 |  |
| Nppb | 18158 | -5.93189 | 0.006673 | GO:0003085,GO:0005179,GO:0005576,GO:0005615 | natriuretic peptide type B |  |
| Sds | 231691 | -5.55356 | 0.000495 | GO:0003941,GO:0004794,GO:0005737,GO:0005739 | serine dehydratase |  |
| Nppa | 230899 | -5.40028 | 0.000163 | GO:0003085,GO:0005102,GO:0005179,GO:0005184 | natriuretic peptide type A |  |
| Tmem179 | 104885 | -4.87355 | 0.008918 | GO:0016020,GO:0016021 | transmembrane protein 179 |  |
| Serpina1d | 20703 | -4.63073 | 0.001089 | GO:0002020,GO:0004866,GO:0004867,GO:0005576 | serine (or cysteine) peptidase inhibitor,  clade A, member 1D |  |
| Mab21l2 | 23937 | -4.58456 | 0.011574 | GO:0001654,GO:0005634,GO:0005737,GO:0007275,GO:0008284,GO:0010172 | mab-21-like 2 |  |
| Prl8a9 | 67310 | -4.26563 | 0.035554 | GO:0005148,GO:0005179,GO:0005576,GO:0005615 | prolactin family8,  subfamily a, member 9 |  |
| Soga3 | 67412 | -4.25064 | 0.042563 | GO:0005615,GO:0010506,GO:0016020,GO:0016021 | SOGA family member 3 |  |
| Gm29721 | 101055745 | -4.20163 | 0.037132 |  |  |  |
| Gpr1 | 241070 | -4.16962 | 0.035883 | GO:0004930,GO:0005654,GO:0005886,GO:0005887 | G protein-coupled receptor 1 |  |
| Ptgds | 19215 | -4.11196 | 0.000176 | GO:0001516,GO:0004667,GO:0005501,GO:0005504 | prostaglandin D2 synthase (brain) |  |
| Cst13 | 69294 | -4.11171 | 0.03933 | GO:0004869,GO:0005576,GO:0005737,GO:0010466,GO:0030414 | cystatin 13 |  |
| Dleu7 | 239133 | -4.07437 | 0.011325 |  | deleted in lymphocytic leukemia, 7 |  |
| Gm2446 | 100039830 | -3.95927 | 0.002181 |  |  |  |
| Cdh18 | 320865 | -3.70063 | 0.018635 | GO:0000902,GO:0005509,GO:0005912,GO:0007043 | cadherin 18 |  |
| Sult1e1 | 20860 | -3.63036 | 0.000247 | GO:0004062,GO:0004304,GO:0005496,GO:0005737, | sulfotransferase family 1E, member 1 |  |
| Gpr87 | 84111 | -3.62966 | 0.032341 | GO:0004930,GO:0005886,GO:0007165,GO:0007186 | G protein-coupled receptor 87 |  |
| Sh2d7 | 244885 | -3.62072 | 0.038591 |  | SH2 domain containing 7 |  |
| Nrip2 | 60345 | -3.62037 | 0.002191 | GO:0000122,GO:0004190,GO:0005515,GO:0005634,GO:0005737,GO:0006508,GO:0007219 | nuclear receptor interacting protein 2 |  |
| Rgs13 | 246709 | -3.61746 | 0.024568 | GO:0005515,GO:0005634,GO:0005829,GO:0005886,GO:0007186,GO:0009968,GO:0045744 | regulator of G-protein signaling 13 |  |
| Lypd5 | 76942 | -3.61506 | 0.02657 | GO:0005886,GO:0007160,GO:0016020,GO:0031225,GO:0043236 | Ly6/Plaur domain containing 5 |  |
| Capn11 | 268958 | -3.49678 | 0.047175 | GO:0001669,GO:0004198,GO:0005509,GO:0005737 | calpain 11 |  |
| Psca | 72373 | -3.41249 | 0.023061 | GO:0005886,GO:0016020,GO:0031225,GO:0033130,GO:0070373,GO:0099601 | prostate stem cell antigen |  |
| Opn4 | 30044 | -3.31062 | 0.003306 | GO:0004930,GO:0005502,GO:0005886,GO:0005887 | opsin 4 (melanopsin) |  |
| Ntng1 | 80883 | -3.28464 | 0.026906 | GO:0005515,GO:0005886,GO:0007275,GO:0007399 | netrin G1 |  |
| Aldh3b3 | 73458 | -3.23552 | 0.017675 | GO:0004028,GO:0004029,GO:0005737,GO:0005886 | aldehyde dehydrogenase 3 family, member B3 |  |
| Defb1 | 13214 | -3.17315 | 0.00442 | GO:0002227,GO:0005576,GO:0005615,GO:0006952 | defensin beta 1 |  |
| Hspa1b | 15511 | -3.15091 | 0.000462 | GO:0002199,GO:0005515,GO:0005524,GO:0005622 | heat shock protein 1B |  |
| Hspa1a | 193740 | -3.12607 | 0.000168 | GO:0000723,GO:0001664,GO:0003714,GO:0003725 | heat shock protein 1A |  |
| Gm21976 | 102637808 | -3.00207 | 0.006456 |  |  |  |
| Col9a2 | 12840 | -2.97691 | 0.020495 | GO:0005201,GO:0005576,GO:0005581,GO:0005594 | collagen, type IX, alpha 2 |  |
| Odf4 | 252868 | -2.9531 | 0.035084 | GO:0001520,GO:0005886,GO:0007275,GO:0007283 | outer dense fiber of  sperm tails 4 |  |
| Hand2 | 15111 | -2.93897 | 0.000558 | GO:0000785,GO:0000976,GO:0000977,GO:0000981 | heart and neural crest derivatives expressed 2 |  |
| Ppef1 | 237178 | -2.91141 | 0.009843 | GO:0004721,GO:0004722,GO:0005506,GO:0005509,GO:0005634,GO:0005829 | protein phosphatase with EF hand calcium-binding domain 1 |  |
| Gpat2 | 215456 | -2.82576 | 0.034151 | GO:0003841,GO:0004366,GO:0005515,GO:0005739,GO:0005741,GO:0006072, | glycerol-3-phosphate acyltransferase 2, mitochondrial |  |
| Khdc1a | 368204 | -2.7975 | 0.038178 | GO:0003723,GO:0005515,GO:0005737,GO:0006915, | KH domain containing 1A |  |
| Olfm2 | 244723 | -2.69475 | 9.67E-14 | GO:0005515,GO:0005576,GO:0005634,GO:0005654 | olfactomedin 2 |  |
| Krt17 | 16667 | -2.67553 | 0.00254 | GO:0002009,GO:0005198,GO:0005515,GO:0005737 | keratin 17 |  |
| Cyp1a1 | 13076 | -2.67177 | 1.07E-06 | GO:0002933,GO:0003824,GO:0004497,GO:0005506,GO:0005737,GO:0005739 | cytochrome P450,  family 1, subfamily a, polypeptide 1 |  |
| Slco1a4 | 28250 | -2.67093 | 0.005079 | GO:0005886,GO:0005887,GO:0006811,GO:0006820,GO:0008514,GO:0009925 | solute carrier organic anion transporter family,  member 1a4 |  |
| Unc13c | 208898 | -2.58657 | 0.032767 | GO:0001566,GO:0005509,GO:0005516,GO:0005543 | unc-13 homolog C |  |
| Gm32717 | 102635357 | -2.5007 | 0.031874 | GO:0000398,GO:0000974,GO:0000977,GO:0000981,GO:0005681,GO:0006357 |  |  |
| Hspb3 | 56534 | -2.48221 | 0.031528 | GO:0005634,GO:0005737,GO:0016607 | heat shock protein 3 |  |
| Lrrc10 | 237560 | -2.45517 | 0.009829 | GO:0003779,GO:0005634,GO:0005739,GO:0005856,GO:0030016,GO:0030017,GO:0051393,GO:0055013 | leucine rich repeat containing 10 |  |
| Thrsp | 21835 | -2.41717 | 9.73E-08 | GO:0005515,GO:0005634,GO:0005654,GO:0005737 | thyroid hormone responsive |  |
| 2010003K11Rik | 69861 | -2.41051 | 0.02149 |  | RIKEN cDNA 2010003K11 gene |  |
| Cplx1 | 12889 | -2.40669 | 0.022027 | GO:0000149,GO:0005326,GO:0005737,GO:0006836,GO:0006887,GO:0016079 | complexin 1 |  |
| Adprhl1 | 234072 | -2.27442 | 0.006931 | GO:0000287,GO:0003875,GO:0005096,GO:0006886,GO:0016787,GO:0051725,GO:0090630 | ADP-ribosylhydrolase  like 1 |  |

**Table S3. Top regulated genes in the Diabetes+Acarbose+Infected VS Diabetes+Infected**

| **Gene Name** | **Gene ID** | **log2FoldChange** | **p-value** | **GO ID** | **Gene Description** | |
| --- | --- | --- | --- | --- | --- | --- |
| Top 50 up-regulated genes in Diabetes+Acarbose+Infected VS Diabetes+Infected | | | | | | |
| Opn1mw | 14539 | 4.944962 | 0.019146 | GO:0001750,GO:0004930,GO:0005515,GO:0005886,GO:0005887,GO:0007165 | opsin 1 (cone pigments), medium-wave-sensitive (color blindness, deutan) |  |
| Tspan10 | 208634 | 4.714424 | 0.014699 | GO:0005887,GO:0016020,GO:0016021,GO:0019899,GO:0051604,GO:0072594 | tetraspanin 10 |  |
| Klk1b4 | 18048 | 4.577829 | 0.035742 | GO:0003073,GO:0004175,GO:0004252,GO:0005615 | kallikrein 1-related pepidase b4 |  |
| Prl8a9 | 67310 | 4.450639 | 0.002759 | GO:0005148,GO:0005179,GO:0005576,GO:0005615 | prolactin family8,  subfamily a, member 9 |  |
| Chil4 | 104183 | 4.318044 | 0.005014 | GO:0005576,GO:0005737,GO:0005975,GO:0006032,GO:0006954,GO:0008061 | chitinase-like 4 |  |
| Gm2007 | 102639653 | 4.28342 | 0.048812 |  | predicted gene 2007 |  |
| LOC115489545 | 115489545 | 3.859412 | 0.013321 |  |  |  |
| Apoc4 | 11425 | 3.843678 | 0.003916 | GO:0005576,GO:0006869,GO:0010890,GO:0034361,GO:0034364,GO:0070328 | apolipoprotein C-IV |  |
| Dleu7 | 239133 | 3.798435 | 0.022247 |  | deleted in lymphocytic leukemia, 7 |  |
| Psg16 | 26436 | 3.659767 | 0.017297 | GO:0009986 | pregnancy specific glycoprotein 16 |  |
| Fem1al | 216622 | 3.535078 | 0.030184 | GO:0000151,GO:0005737,GO:0006511,GO:0050728 | fem-1 homolog A like |  |
| Psca | 72373 | 3.238623 | 0.037119 | GO:0005886,GO:0016020,GO:0031225,GO:0033130,GO:0070373,GO:0099601 | prostate stem cell antigen |  |
| Defb20 | 319579 | 3.234587 | 0.031415 | GO:0005576,GO:0006952,GO:0042742,GO:0045087 | defensin beta 20 |  |
| Gm14200 | 115489487 | 3.22773 | 0.004009 |  |  |  |
| Gpat2 | 215456 | 2.941593 | 0.025276 | GO:0003841,GO:0004366,GO:0005515,GO:0005739,GO:0005741,GO:0006072 | glycerol-3-phosphate acyltransferase 2, mitochondrial |  |
| Spem1 | 74288 | 2.867206 | 0.04405 | GO:0005737,GO:0007275,GO:0007283,GO:0007291 | sperm maturation 1 |  |
| Klk1b11 | 16613 | 2.83533 | 0.002237 | GO:0003073,GO:0004175,GO:0004252,GO:0005615 | kallikrein 1-related peptidase b11 |  |
| Serpina1b | 20701 | 2.802789 | 0.035143 | GO:0001701,GO:0002020,GO:0004866,GO:0004867,GO:0005515,GO:0005576 | serine (or cysteine) preptidase inhibitor,  clade A, member 1B |  |
| H2ac8 | 319166 | 2.800474 | 0.023004 | GO:0003677,GO:0005634,GO:0006342,GO:0019899 | H2A clustered histone 8 |  |
| Tpbpb | 116913 | 2.792169 | 0.046704 |  | trophoblast specific  protein beta |  |
| Ccl21b | 100042493 | 2.779442 | 0.021751 | GO:0002548,GO:0005615,GO:0006954,GO:0007186 | chemokine (C-C motif) ligand 21B (leucine) |  |
| Ppef1 | 237178 | 2.761402 | 0.020775 | GO:0004721,GO:0004722,GO:0005506,GO:0005509,GO:0005634,GO:0005829 | protein phosphatase with EF hand calcium-binding domain 1 |  |
| Ido1 | 15930 | 2.69292 | 0.017656 | GO:0002376,GO:0002534,GO:0002666,GO:0002678 | indoleamine 2,3-dioxygenase 1 |  |
| Duoxa2 | 66811 | 2.380076 | 0.023738 | GO:0005783,GO:0005789,GO:0005829,GO:0005886 | dual oxidase maturation factor 2 |  |
| Prss2 | 22072 | 2.37487 | 0.021622 | GO:0004252,GO:0005509,GO:0005576,GO:0005615 | protease, serine 2 |  |
| Rnf182 | 328234 | 2.207879 | 0.043096 | GO:0004842,GO:0005737,GO:0016020,GO:0016021,GO:0016567,GO:0016740,GO:0046872 | ring finger protein 182 |  |
| Igdcc3 | 19289 | 2.146641 | 0.0432 | GO:0016020,GO:0016021,GO:0050885 | immunoglobulin superfamily,  DCC subclass, member 3 |  |
| Il17a | 16171 | 2.129318 | 0.009936 | GO:0002225,GO:0002250,GO:0002376,GO:0005125 | interleukin 17A |  |
| Sox15 | 20670 | 1.994732 | 0.037022 | GO:0000122,GO:0000978,GO:0000981,GO:0003677 | SRY (sex determining region Y)-box 15 |  |
| H4c4 | 319156 | 1.984628 | 0.023835 | GO:0000228,GO:0000786,GO:0005634,GO:0006334 | H4 clustered histone 4 |  |
| Serpina10 | 217847 | 1.830007 | 0.005137 | GO:0004867,GO:0005576,GO:0005615,GO:0007596,GO:0007599,GO:0008201,GO:0010466,GO:0010951,GO:0030414 | serine (or cysteine) peptidase inhibitor, clade A (alpha-1 antiproteinase, antitrypsin), member 10 |  |
| Frmpd3 | 245643 | 1.797294 | 0.038178 |  | FERM and PDZ domain containing 3 |  |
| Crisp2 | 22024 | 1.789147 | 0.026365 | GO:0005576,GO:0005615,GO:0098609 | cysteine-rich secretory protein 2 |  |
| Lonrf2 | 381338 | 1.696929 | 0.009155 |  | LON peptidase N-terminal domain and ring finger 2 |  |
| Rnase2b | 54159 | 1.692963 | 0.010575 | GO:0002227,GO:0003676,GO:0004518,GO:0004519,GO:0004522,GO:0004540,GO:0005615,GO:0006935,GO:0016787,GO:0016829 | ribonuclease, RNase A family, 2B  (liver, eosinophil-derived neurotoxin) |  |
| Scrt1 | 170729 | 1.67331 | 0.036544 | GO:0000122,GO:0000977,GO:0000978,GO:0000981 | scratch family zinc finger 1 |  |
| 2210418O10Rik | 100504263 | 1.662744 | 0.039498 |  | RIKEN cDNA  2210418O10 gene |  |
| Galr3 | 14429 | 1.624794 | 0.042224 | GO:0004930,GO:0004966,GO:0005886,GO:0005887 | galanin receptor 3 |  |
| Trim69 | 70928 | 1.554851 | 0.034349 | GO:0004842,GO:0005634,GO:0005737,GO:0006915 | tripartite  motif-containing 69 |  |
| Ccl8 | 20307 | 1.540024 | 0.025001 | GO:0002548,GO:0005125,GO:0005576,GO:0005615 | chemokine (C-C motif) ligand 8 |  |
| Hsd17b1 | 15485 | 1.524731 | 0.033433 | GO:0004303,GO:0005496,GO:0005737,GO:0005829 | hydroxysteroid (17-beta) dehydrogenase 1 |  |
| Spink4 | 20731 | 1.505421 | 0.000622 | GO:0004867,GO:0005576,GO:0010466,GO:0030414 | serine peptidase inhibitor, Kazal type 4 |  |
| Cenps | 69928 | 1.49966 | 0.030168 | GO:0000712,GO:0000775,GO:0000776,GO:0003677 | centromere protein S |  |
| Shisa8 | 435145 | 1.460038 | 0.005792 | GO:0014069,GO:0016020,GO:0016021,GO:0032281 | shisa family member 8 |  |
| Nat8f5 | 69049 | 1.445425 | 0.008551 | GO:0001702,GO:0005615,GO:0005783,GO:0005794 | N-acetyltransferase 8 (GCN5-related) family member 5 |  |
| Rpl39l | 68172 | 1.431615 | 0.049908 | GO:0022625 | ribosomal protein L39-like |  |
| Cyp4f14 | 64385 | 1.408397 | 0.004911 | GO:0004497,GO:0005506,GO:0005783,GO:0006690 | cytochrome P450, family 4, subfamily f, polypeptide 14 |  |
| Tmem266 | 244886 | 1.388949 | 0.042165 | GO:0005216,GO:0005829,GO:0005886,GO:0005887 | transmembrane protein 266 |  |
| Hspa1b | 15511 | 1.31476 | 0.019512 | GO:0002199,GO:0005515,GO:0005524,GO:0005622 | heat shock protein 1B |  |
| Vsig1 | 78789 | 1.284546 | 0.043223 | GO:0003382,GO:0005886,GO:0016020,GO:0016021,GO:0016323,GO:0030277 | V-set and immunoglobulin domain containing 1 |  |
| Top 50 down-regulated genes in Diabetes+Acarbose+Infected VS Diabetes+Infected | | | | | | |
| Ngp | 18054 | -6.55291 | 2.45E-05 | GO:0004869,GO:0005576,GO:0005615,GO:0006952 | neutrophilic granule protein |  |
| Fpr3 | 14294 | -6.2278 | 6.86E-06 | GO:0002430,GO:0004875,GO:0004930,GO:0004982 | formyl peptide receptor 3 |  |
| Btnl10 | 192194 | -5.90452 | 0.000691 | GO:0001817,GO:0005102,GO:0009897,GO:0016020,GO:0016021,GO:0050852 | butyrophilin-like 10 |  |
| Gm6551 | 625060 | -5.55132 | 0.043796 |  | predicted gene 6551 |  |
| Rhd | 19746 | -5.53507 | 0.000475 | GO:0005886,GO:0005887,GO:0008519,GO:0015696 | Rh blood group, D antigen |  |
| Saa2 | 20209 | -5.53479 | 0.009604 | GO:0001664,GO:0005515,GO:0005576,GO:0005881 | serum amyloid A 2 |  |
| Itgb2l | 16415 | -5.03201 | 0.0001 | GO:0001540,GO:0005178,GO:0005886,GO:0005925 | integrin beta 2-like |  |
| Ms4a3 | 170813 | -4.97662 | 0.011214 | GO:0005515,GO:0005737,GO:0016020,GO:0016021,GO:0051726 | membrane-spanning 4-domains, subfamily A, member 3 |  |
| Camp | 12796 | -4.87075 | 0.000377 | GO:0001530,GO:0001878,GO:0001934,GO:0002227 | cathelicidin antimicrobial peptide |  |
| Rag1 | 19373 | -4.55013 | 0.040884 | GO:0002250,GO:0002331,GO:0003677,GO:0003824 | recombination activating 1 |  |
| Slc15a5 | 277898 | -4.35204 | 0.017145 | GO:0015031,GO:0015293,GO:0015833,GO:0016020 | solute carrier family 15, member 5 |  |
| Kel | 23925 | -4.25965 | 0.003746 | GO:0004175,GO:0004222,GO:0005654,GO:0005886 | Kell blood group |  |
| Trim58 | 216781 | -4.21715 | 0.003972 | GO:0000209,GO:0005737,GO:0006511,GO:0008270 | tripartite motif-containing 58 |  |
| Prss34 | 328780 | -3.99963 | 0.000116 | GO:0004252,GO:0005539,GO:0005615,GO:0006508 | protease, serine 34 |  |
| Alox8 | 11688 | -3.87222 | 0.000799 | GO:0005506,GO:0005509,GO:0005737,GO:0005829 | arachidonate 8-lipoxygenase |  |
| Olfm4 | 380924 | -3.75946 | 0.002188 | GO:0005198,GO:0005515,GO:0005576,GO:0005615 | olfactomedin 4 |  |
| Tspo2 | 70026 | -3.68287 | 5.95E-05 | GO:0005783,GO:0005886,GO:0015485,GO:0016020,GO:0016021,GO:0031090 | translocator protein 2 |  |
| Ush1c | 72088 | -3.63298 | 0.013918 | GO:0000086,GO:0001750,GO:0001917,GO:0002142 | USH1 protein network component harmonin |  |
| Otud7a | 170711 | -3.61543 | 0.018401 | GO:0003677,GO:0004843,GO:0005634,GO:0005737 | OTU domain containing 7A |  |
| Prok2 | 50501 | -3.56877 | 0.016842 | GO:0000187,GO:0001525,GO:0001664,GO:0001935 | prokineticin 2 |  |
| Slc4a1 | 20533 | -3.55508 | 0.000228 | GO:0003779,GO:0005452,GO:0005515,GO:0005886 | solute carrier family 4 (anion exchanger),  member 1 |  |
| Myot | 58916 | -3.54862 | 0.032781 | GO:0003779,GO:0005737,GO:0005856,GO:0005886 | myotilin |  |
| Hemgn | 93966 | -3.41286 | 0.000692 | GO:0005634,GO:0005654,GO:0007275,GO:0030154,GO:0045667 | hemogen |  |
| Cartpt | 27220 | -3.38819 | 0.017389 | GO:0000186,GO:0001678,GO:0005184,GO:0005576 | CART prepropeptide |  |
| Ly6g6f | 433099 | -3.38334 | 0.004491 |  | lymphocyte antigen 6 complex, locus G6F |  |
| Rhag | 19743 | -3.21818 | 0.007922 | GO:0005886,GO:0005887,GO:0006873,GO:0008519 | Rhesus blood group-associated A glycoprotein |  |
| Gm34362 | 102637593 | -3.20703 | 0.048069 |  |  |  |
| Cbln3 | 56410 | -3.18473 | 0.022449 | GO:0005515,GO:0005576,GO:0005615,GO:0005783,GO:0005794,GO:0030054 | cerebellin 3 precursor protein |  |
| Wee2 | 381759 | -3.16889 | 0.041376 | GO:0000166,GO:0000278,GO:0000287,GO:0004672 | WEE1 homolog 2 (S. pombe) |  |
| C1rb | 667277 | -3.1595 | 0.002958 | GO:0002376,GO:0004252,GO:0005509,GO:0005615 | complement component 1, r subcomponent B |  |
| Gm14288 | 13999 | -3.1357 | 0.038734 |  | predicted gene 14288 |  |
| 1300017J02Rik | 71775 | -3.09154 | 0.010452 | GO:0004857,GO:0005506,GO:0005576,GO:0005615 | RIKEN cDNA  1300017J02 gene |  |
| Slfn14 | 237890 | -3.07352 | 0.001324 | GO:0004518,GO:0004519,GO:0004521,GO:0005634 | schlafen 14 |  |
| Chst9 | 71367 | -3.05342 | 0.041651 | GO:0001537,GO:0005794,GO:0005975,GO:0006790,GO:0008146,GO:0016020 | carbohydrate (N-acetylgalactosamine 4-0) sulfotransferase 9 |  |
| Olfr1396 | 258334 | -3.05319 | 0.017249 | GO:0004984,GO:0007186,GO:0007608,GO:0016021 | olfactory receptor 1396 |  |
| Car1 | 12346 | -3.01821 | 0.020789 | GO:0004064,GO:0004089,GO:0005737,GO:0006730 | carbonic anhydrase 1 |  |
| Mpo | 17523 | -3.01712 | 0.037724 | GO:0001878,GO:0002149,GO:0002679,GO:0004601 | myeloperoxidase |  |
| Trim10 | 19824 | -2.97345 | 0.000168 | GO:0005515,GO:0005737,GO:0008270,GO:0010468 | tripartite  motif-containing 10 |  |
| Apof | 103161 | -2.93466 | 0.012825 | GO:0005576,GO:0005615,GO:0006629,GO:0006641 | apolipoprotein F |  |
| Zim1 | 22776 | -2.79296 | 0.038494 | GO:0000978,GO:0000981,GO:0005634,GO:0006357 | zinc finger, imprinted 1 |  |
| Pirt | 193003 | -2.74577 | 0.04087 | GO:0005547,GO:0005886,GO:0009408,GO:0016020,GO:0016021,GO:0044325,GO:0048015,GO:0048266,GO:1902936,GO:2001259 | phosphoinositide-interacting regulator of transient receptor potential channels |  |
| Spta1 | 20739 | -2.74111 | 0.000313 | GO:0002260,GO:0003779,GO:0005509,GO:0005515 | spectrin alpha,  erythrocytic 1 |  |
| Cd5l | 11801 | -2.72861 | 0.000913 | GO:0002376,GO:0004252,GO:0005044,GO:0005576 | CD5 antigen-like |  |
| Add2 | 11519 | -2.71482 | 0.00705 | GO:0003779,GO:0005200,GO:0005516,GO:0005737 | adducin 2 (beta) |  |
| Slc51a | 106407 | -2.70066 | 0.022793 | GO:0005215,GO:0005515,GO:0005783,GO:0005789 | solute carrier family 51, alpha subunit |  |
| Chil5 | 229687 | -2.69822 | 0.008277 | GO:0005576,GO:0006032,GO:0008061 | chitinase-like 5 |  |
| Ifitm6 | 213002 | -2.56131 | 0.024446 | GO:0005886,GO:0034341,GO:0035455,GO:0035456 | interferon induced transmembrane protein 6 |  |
| Gypa | 14934 | -2.48793 | 0.00342 | GO:0005887,GO:0009897,GO:0016020,GO:0016021,GO:0042802,GO:0047484 | glycophorin A |  |
| Apol11b | 328563 | -2.42958 | 0.000479 | GO:0008289 | apolipoprotein L 11b |  |
| Ggt1 | 14598 | -2.39701 | 0.003209 | GO:0000048,GO:0002682,GO:0002951,GO:0005615 | gamma-glutamyltransferase 1 |  |

**Table S4. Top regulated genes in the Ctrl+Acarbose+Infected VS Ctrl+Infected**

| **Gene Name** | **Gene ID** | **log2FoldChange** | **p-value** | **GO ID** | **Gene Description** | |
| --- | --- | --- | --- | --- | --- | --- |
| Top 42 up-regulated genes in Ctrl+Acarbose+Infected VS Ctrl+Infected | | | | | | |
| Chil4 | 104183 | 7.866712 | 2.93E-07 | GO:0005576,GO:0005737,GO:0005975,GO:0006032 | chitinase-like 4 |  |
| Prok2 | 50501 | 6.116823 | 0.043421 | GO:0000187,GO:0001525,GO:0001664,GO:0001935 | prokineticin 2 |  |
| Clca1 | 23844 | 6.078287 | 7.21E-07 | GO:0005229,GO:0005254,GO:0005576,GO:0005887 | chloride channel accessory 1 |  |
| Umodl1 | 52020 | 5.112725 | 0.000427 | GO:0005201,GO:0005509,GO:0005576,GO:0005615 | uromodulin-like 1 |  |
| Gm34362 | 102637593 | 4.9967 | 0.000851 |  |  |  |
| Neurod4 | 11923 | 4.697814 | 0.003188 | GO:0000978,GO:0000981,GO:0001764,GO:0003677 | neurogenic differentiation 4 |  |
| Fibcd1 | 98970 | 4.581445 | 0.013554 | GO:0005102,GO:0005615,GO:0007155,GO:0008061 | fibrinogen C domain containing 1 |  |
| Ctsm | 64139 | 4.369723 | 0.010492 | GO:0004197,GO:0005615,GO:0005764,GO:0006508 | cathepsin M |  |
| Khdc1b | 98582 | 4.135936 | 0.015539 | GO:0003723,GO:0005515,GO:0005737,GO:0006919,GO:0042802 | KH domain containing 1B |  |
| Tex44 | 71863 | 3.971041 | 0.024164 | GO:0005737 | testis expressed 44 |  |
| Ndst4 | 64580 | 3.807537 | 0.032625 | GO:0003824,GO:0005794,GO:0008146,GO:0008152,GO:0015014,GO:0015016 | N-deacetylase/N-sulfotransferase (heparin glucosaminyl) 4 |  |
| 6030468B19Rik | 77727 | 3.798473 | 0.010627 | GO:0002250,GO:0002313,GO:0002376,GO:0004888 | RIKEN cDNA 6030468B19 gene |  |
| Aire | 11634 | 3.78116 | 0.004347 | GO:0000977,GO:0002458,GO:0002509,GO:0003677,GO:0003682,GO:0005622,GO:0005634,GO:0005737,GO:0006355,GO:0006959 | autoimmune regulator (autoimmune polyendocrinopathy candidiasis ectodermal dystrophy) |  |
| Noto | 384452 | 3.643051 | 0.020176 | GO:0000978,GO:0000981,GO:0001947,GO:0003677 | notochord homeobox |  |
| Gm10591 | 100504239 | 3.451679 | 0.008181 |  | predicted gene 10591 |  |
| Tmem229a | 319832 | 3.433958 | 0.026035 | GO:0016020,GO:0016021 | transmembrane  protein 229A |  |
| Car6 | 12353 | 3.381975 | 0.01297 | GO:0001580,GO:0004089,GO:0005576,GO:0005615 | carbonic anhydrase 6 |  |
| Prl7a2 | 19114 | 2.977482 | 0.027285 | GO:0005148,GO:0005179,GO:0005576,GO:0005615 | prolactin family 7, subfamily a, member 2 |  |
| Lrcol1 | 381667 | 2.812536 | 0.001294 | GO:0005576,GO:0007586,GO:0008047,GO:0016042,GO:0032094 | leucine rich colipase-like 1 |  |
| Tmem233 | 545798 | 2.810606 | 0.021007 | GO:0016020,GO:0016021 | transmembrane protein 233 |  |
| Slc6a3 | 13162 | 2.651544 | 0.019439 | GO:0001504,GO:0002020,GO:0005102,GO:0005326,GO:0005330,GO:0005334,GO:0005515,GO:0005886 | solute carrier family 6 (neurotransmitter transporter, dopamine), member 3 |  |
| Hsd17b13 | 243168 | 2.592324 | 0.04337 | GO:0005783,GO:0005811,GO:0016229,GO:0016491,GO:0016616,GO:0046889 | hydroxysteroid (17-beta) dehydrogenase 13 |  |
| Serpina11 | 380780 | 2.499315 | 0.002924 | GO:0004867,GO:0005576,GO:0005615,GO:0010466,GO:0010951,GO:0030414 | serine (or cysteine) peptidase inhibitor, clade A (alpha-1 antiproteinase, antitrypsin), member 11 |  |
| Tktl1 | 83553 | 2.376867 | 0.037641 | GO:0003824,GO:0004802,GO:0005634,GO:0005737 | transketolase-like 1 |  |
| H3c11 | 319153 | 2.371265 | 0.02047 | GO:0000228,GO:0000786,GO:0005634,GO:0006334 | H3 clustered histone 11 |  |
| Zkscan16 | 100041581 | 2.348018 | 0.000587 | GO:0000978,GO:0000981,GO:0005634,GO:0006357 | zinc finger with KRAB and SCAN domains 16 |  |
| Serpinb2 | 18788 | 2.311485 | 0.000589 | GO:0004867,GO:0005576,GO:0005615,GO:0005737,GO:0010466,GO:0010951,GO:0030414,GO:0042060,GO:0043066 | serine (or cysteine) peptidase inhibitor,  clade B, member 2 |  |
| Cd300ld5 | 100043125 | 2.010825 | 0.011791 |  | CD300 molecule like family member D5 |  |
| A530021J07Rik | 330578 | 1.999822 | 0.042257 |  | Riken cDNA A530021J07 gene |  |
| Gm2102 | 100039210 | 1.741157 | 0.039302 |  | predicted gene 2102 |  |
| 1700018F24Rik | 69396 | 1.70404 | 0.019147 |  | RIKEN cDNA 1700018F24 gene |  |
| Clec4a4 | 474145 | 1.512308 | 0.01971 | GO:0097367 | C-type lectin domain family 4, member a4 |  |
| Fam177a2 | 100101807 | 1.378644 | 0.018326 |  | family with sequence similarity 177 member A2 |  |
| 6430550D23Rik | 320095 | 1.361813 | 0.006174 | GO:0003836 | RIKEN cDNA 6430550D23 gene |  |
| Irgc1 | 210145 | 1.335212 | 0.004789 | GO:0000166,GO:0003924,GO:0005525,GO:0005789,GO:0006952,GO:0016020,GO:0016787,GO:0035458 | immunity-related GTPase family, cinema 1 |  |
| Ccna1 | 12427 | 1.277949 | 0.03951 | GO:0000079,GO:0000307,GO:0005515,GO:0005634 | cyclin A1 |  |
| Il5ra | 16192 | 1.258742 | 0.015321 | GO:0002437,GO:0004896,GO:0009897,GO:0016020 | interleukin 5 receptor, alpha |  |
| Gm31493 | 102633740 | 1.248012 | 0.007962 |  |  |  |
| Alox15 | 11687 | 1.197972 | 0.015681 | GO:0001503,GO:0002820,GO:0004052,GO:0005506 | arachidonate 15-lipoxygenase |  |
| Htr2c | 15560 | 1.10179 | 0.001432 | GO:0001587,GO:0001662,GO:0004930,GO:0004993 | 5-hydroxytryptamine (serotonin) receptor 2C |  |
| Slco5a1 | 240726 | 1.097181 | 0.002716 | GO:0005886,GO:0005887,GO:0015347,GO:0043231,GO:0043252 | solute carrier organic anion transporter family,  member 5A1 |  |
| B3gnt6 | 272411 | 1.003839 | 0.049331 | GO:0005794,GO:0006486,GO:0008375,GO:0008376,GO:0008378,GO:0008532,GO:0016020,GO:0016021 | UDP-GlcNAc:betaGal beta-1,3-N-acetylglucosaminyltransferase 6 (core 3 synthase) |  |
| Top 50 down-regulated genes in Diabetes+Acarbose+Infected VS Diabetes+Infected | | | | | | |
| Elovl3 | 12686 | -20.7984 | 6.64E-12 | GO:0005783,GO:0006629,GO:0006631,GO:0006633,GO:0009922,GO:0016020,GO:0016021,GO:0016740 | elongation of very long chain fatty acids (FEN1/Elo2, SUR4/Elo3, yeast)-like 3 |  |
| Bmp10 | 12154 | -9.97118 | 0.000985 | GO:0001822,GO:0005125,GO:0005179,GO:0005576 | bone morphogenetic protein 10 |  |
| Nppb | 18158 | -8.41099 | 9.18E-05 | GO:0003085,GO:0005179,GO:0005576,GO:0005615 | natriuretic peptide type B |  |
| Gm4779 | 102634296 | -6.21657 | 0.000236 |  | predicted gene 4779 |  |
| Nppa | 230899 | -5.38917 | 0.000307 | GO:0003085,GO:0005102,GO:0005179,GO:0005184 | natriuretic peptide type A |  |
| Tmem179 | 104885 | -4.87585 | 0.008159 | GO:0016020,GO:0016021 | transmembrane protein 179 |  |
| Mslnl | 328783 | -4.8208 | 0.000213 | GO:0007155,GO:0007160,GO:0009986,GO:0016020,GO:0016021 | mesothelin-like |  |
| Nmrk2 | 69564 | -4.72835 | 0.011071 | GO:0000166,GO:0005515,GO:0005524,GO:0005622 | nicotinamide riboside kinase 2 |  |
| Retnlb | 57263 | -4.61504 | 0.034357 | GO:0005179,GO:0005576,GO:0005615,GO:0009617 | resistin like beta |  |
| Cck | 12424 | -4.15891 | 0.019355 | GO:0001764,GO:0001836,GO:0005179,GO:0005184 | cholecystokinin |  |
| Emilin3 | 280635 | -4.11403 | 0.044536 | GO:0005576,GO:0005737,GO:0042802 | elastin microfibril interfacer 3 |  |
| Mab21l2 | 23937 | -4.00893 | 0.015807 | GO:0001654,GO:0005634,GO:0005737,GO:0007275,GO:0008284,GO:0010172,GO:0043010 | mab-21-like 2 |  |
| 8030474K03Rik | 382231 | -3.98292 | 0.022955 |  | RIKEN cDNA 8030474K03 gene |  |
| Chodl | 246048 | -3.96584 | 0.031948 | GO:0005515,GO:0005540,GO:0005737,GO:0005813 | chondrolectin |  |
| Nrsn1 | 22360 | -3.84881 | 0.034308 | GO:0007399,GO:0016020,GO:0016021,GO:0030133 | neurensin 1 |  |
| Gm7972 | 666190 | -3.78953 | 0.010237 |  | predicted gene 7972 |  |
| Ccdc110 | 212392 | -3.77822 | 0.020505 | GO:0005634,GO:0005856 | coiled-coil domain containing 110 |  |
| Ush1g | 16470 | -3.74287 | 0.008164 | GO:0001917,GO:0005515,GO:0005737,GO:0005856 | USH1 protein network component sans |  |
| Fmr1nb | 207854 | -3.73764 | 0.02671 | GO:0016020,GO:0016021 | Fmr1 neighbor |  |
| Cdh18 | 320865 | -3.69967 | 0.021055 | GO:0000902,GO:0005509,GO:0005912,GO:0007043 | cadherin 18 |  |
| Cysrt1 | 67859 | -3.4864 | 0.008674 | GO:0042802 | cysteine rich tail 1 |  |
| Dppa3 | 73708 | -3.41056 | 0.016536 | GO:0001939,GO:0001940,GO:0005515,GO:0005634 | developmental pluripotency-associated 3 |  |
| Slc7a9 | 30962 | -3.36018 | 0.032822 | GO:0003333,GO:0005515,GO:0005886,GO:0006865,GO:0015171,GO:0015175,GO:0015179,GO:0015184 | solute carrier family 7 (cationic amino acid transporter, y+ system), member 9 |  |
| Hpca | 15444 | -3.35412 | 0.015635 | GO:0003779,GO:0005509,GO:0005515,GO:0005737 | hippocalcin |  |
| Chrna3 | 110834 | -3.34598 | 0.006808 | GO:0004888,GO:0005216,GO:0005230,GO:0005515,GO:0005886,GO:0005887 | cholinergic receptor, nicotinic, alpha polypeptide 3 |  |
| Orm2 | 18406 | -3.3257 | 0.020186 | GO:0002682,GO:0005576,GO:0005615,GO:0006953 | orosomucoid 2 |  |
| Kcnt1 | 227632 | -3.21884 | 0.027555 | GO:0005228,GO:0005267,GO:0005886,GO:0006811 | potassium channel, subfamily T, member 1 |  |
| Il23a | 83430 | -3.19587 | 0.03081 | GO:0001916,GO:0002230,GO:0002376,GO:0002827 | interleukin 23, alpha subunit p19 |  |
| Hspb3 | 56534 | -3.05094 | 0.0121 | GO:0005634,GO:0005737,GO:0016607 | heat shock protein 3 |  |
| Ptgds | 19215 | -2.99907 | 0.003022 | GO:0001516,GO:0004667,GO:0005501,GO:0005504 | prostaglandin D2 synthase (brain) |  |
| Tnc | 21923 | -2.99197 | 0.003286 | GO:0001968,GO:0005201,GO:0005576,GO:0005604 | tenascin C |  |
| Cxcl5 | 20311 | -2.95142 | 0.02368 | GO:0001776,GO:0005125,GO:0005576,GO:0005615 | chemokine (C-X-C motif) ligand 5 |  |
| Hspa1b | 15511 | -2.74413 | 0.000985 | GO:0002199,GO:0005515,GO:0005524,GO:0005622 | heat shock protein 1B |  |
| Cartpt | 27220 | -2.70145 | 0.025124 | GO:0000186,GO:0001678,GO:0005184,GO:0005576 | CART prepropeptide |  |
| Gm34595 | 102637899 | -2.70088 | 0.032342 |  |  |  |
| Hspa1a | 193740 | -2.61968 | 0.00119 | GO:0000723,GO:0001664,GO:0003714,GO:0003725 | heat shock protein 1A |  |
| Ccl7 | 20306 | -2.60629 | 0.001732 | GO:0002548,GO:0005125,GO:0005576,GO:0005615 | chemokine (C-C motif) ligand 7 |  |
| Slc6a12 | 14411 | -2.59866 | 0.012084 | GO:0005332,GO:0005887,GO:0006836,GO:0008028,GO:0009992,GO:0015293,GO:0015718,GO:0016020, | solute carrier family 6 (neurotransmitter transporter, betaine/GABA), member 12 |  |
| Mroh7 | 381538 | -2.58078 | 0.000762 | GO:0016020,GO:0016021 | maestro heat-like repeat family member 7 |  |
| Klb | 83379 | -2.49798 | 0.004323 | GO:0004553,GO:0005104,GO:0005515,GO:0005783 | klotho beta |  |
| Gdf3 | 14562 | -2.48252 | 0.041291 | GO:0001501,GO:0001654,GO:0001701,GO:0002021 | growth differentiation factor 3 |  |
| Tnni1 | 21952 | -2.47601 | 0.001096 | GO:0003009,GO:0003779,GO:0005861,GO:0006936 | troponin I, skeletal, slow 1 |  |
| Mcidas | 622408 | -2.4703 | 0.001624 | GO:0003713,GO:0005634,GO:0006275,GO:0007049,GO:0007346,GO:0008156,GO:0016604,GO:0030030 | multiciliate differentiation and DNA synthesis associated cell cycle protein |  |
| Cacna2d2 | 56808 | -2.4678 | 0.012818 | GO:0005244,GO:0005245,GO:0005262,GO:0005891,GO:0006811,GO:0006816 | calcium channel, voltage-dependent, alpha 2/delta subunit 2 |  |
| Sectm1a | 209588 | -2.43887 | 0.005947 | GO:0005125,GO:0005576,GO:0005886,GO:0006955,GO:0016020,GO:0016021 | secreted and transmembrane 1A |  |
| Ccl2 | 20296 | -2.42524 | 0.008261 | GO:0001525,GO:0001664,GO:0001912,GO:0001938 | chemokine (C-C motif) ligand 2 |  |
| Aldh3b3 | 73458 | -2.42001 | 0.048106 | GO:0004028,GO:0004029,GO:0005737,GO:0005886 | aldehyde dehydrogenase 3 family, member B3 |  |
| Adamts4 | 240913 | -2.36666 | 0.006591 | GO:0002020,GO:0004222,GO:0005515,GO:0005576,GO:0005615,GO:0006508,GO:0008233,GO:0008237,GO:0016607,GO:0016787 | a disintegrin-like and metallopeptidase (reprolysin type) with thrombospondin type 1 motif, 4 |  |
| Hand2 | 15111 | -2.34982 | 0.003276 | GO:0000785,GO:0000976,GO:0000977,GO:0000981 | heart and neural crest derivatives expressed 2 |  |
| LOC115488529 | 115488529 | -2.32081 | 0.04251 |  |  |  |
